# Exposure to food additive mixtures in 106,000 French adults from the NutriNet-Santé cohort

**DOI:** 10.1038/s41598-021-98496-6

**Published:** 2021-10-04

**Authors:** Eloi Chazelas, Nathalie Druesne-Pecollo, Younes Esseddik, Fabien Szabo de Edelenyi, Cédric Agaesse, Alexandre De Sa, Rebecca Lutchia, Pauline Rebouillat, Bernard Srour, Charlotte Debras, Gaëlle Wendeu-Foyet, Inge Huybrechts, Fabrice Pierre, Xavier Coumoul, Chantal Julia, Emmanuelle Kesse-Guyot, Benjamin Allès, Pilar Galan, Serge Hercberg, Mélanie Deschasaux-Tanguy, Mathilde Touvier

**Affiliations:** 1 Sorbonne Paris Nord University, Inserm U1153, Inrae U1125, Cnam, Nutritional Epidemiology Research Team (EREN), Epidemiology and Statistics Research Center, University of Paris (CRESS), SMBH, Paris 13, 74 rue Marcel Cachin, 93017 Bobigny, Cedex, France; 2 French Network for Nutrition And Cancer Research (NACRe Network), Jouy-en-Josas, France; 3 International Agency for Research On Cancer, World Health Organization, Lyon, France; 4 Toxalim (Research Centre in Food Toxicology), Université de Toulouse, INRAE, ENVT, INP-Purpan, UPS, Toulouse, France; 5 UMR-S1124, Institut national de la santé et de la recherché médicale (Inserm), T3S, Toxicologie Environnementale, Cibles thérapeutiques, Signalisation cellulaire et Biomarqueurs, Paris, France; 6 Public Health Department, Avicenne Hospital, AP-HP, Bobigny, France; 7 Université de Paris, Paris, France

**Keywords:** Nutrition, Statistical methods

## Abstract

Food additives (e.g. artificial sweeteners, emulsifiers, dyes, etc.) are ingested by billions of individuals daily. Some concerning results, mainly derived from animal and/or cell-based experimental studies, have recently emerged suggesting potential detrimental effects of several widely consumed additives. Profiles of additive exposure as well as the potential long-term impact of multiple exposure on human health are poorly documented. This work aimed to estimate the usual intake of food additives among participants of the French NutriNet-Santé cohort and to identify and describe profiles of exposure (single substances and mixtures). Overall, 106,489 adults from the French NutriNet-Santé cohort study (2009-ongoing) were included. Consumption of 90 main food additives was evaluated using repeated 24 h dietary records including information on brands of commercial products. Qualitative information (as presence/absence) of each additive in food products was determined using 3 large-scale composition databases (OQALI, Open Food Facts, GNPD), accounting for the date of consumption of the product. Quantitative ingested doses were estimated using a combination of laboratory assays on food matrixes (n = 2677) and data from EFSA and JECFA. Exposure was estimated in mg per kg of body weight per day. Profiles of exposure to food additive mixtures were extracted using Non-negative Matrix Factorization (NMF) followed by k-means clustering as well as Graphical Lasso. Sociodemographic and dietary comparison of clusters of participants was performed by Chi-square tests or linear regressions. Data were weighted according to the national census. Forty-eight additives were consumed by more than 10% of the participants, with modified starches and citric acid consumed by more than 90%. The top 50 also included several food additives for which potential adverse health effects have been suggested by recent experimental studies: lecithins (86.6% consumers), mono- and diglycerides of fatty acids (78.1%), carrageenan (77.5%), sodium nitrite (73.9%), di-, tri- and polyphosphates (70.1%), potassium sorbate (65.8%), potassium metabisulphite (44.8%), acesulfame K (34.0%), cochineal (33.9%), potassium nitrate (31.6%), sulfite ammonia caramel (28.8%), bixin (19.5%), monosodium glutamate (15.1%) and sucralose (13.5%). We identified and described five clusters of participants more specifically exposed to five distinct additive mixtures and one additional cluster gathering participants with overall low additive exposure. Food additives, including several for which health concerns are currently debated, were widely consumed in this population-based study. Furthermore, main mixtures of additives were identified. Their health impact and potential cocktail effects should be explored in future epidemiological and experimental studies.

## Introduction

Food additives are substances intentionally added to foods during processing, treatment, packaging, transport, or storage. They are used for various technological, sensory and nutritional purposes, such as prolonging shelf life, sweetening, modifying or stabilizing consistency, enhancing taste, and enhancing or preserving color^1^. With a global market exceeding $64 billion, dozens of food additives are ingested daily by billions of individuals worldwide. At the international level, the Codex General Standard for Food Additives (GSFA, Codex STAN 192–1995^2^) sets out the conditions for the use of food additives in food products. In Europe, they represent about 330 authorized compounds under the Regulation (EC) No 1333/2008, and their toxicity is evaluated by the European Food Safety Authority (EFSA). Despite the considerable amount of work for literature review and collective expertise performed by national and international institutions, these evaluations can only be based on currently available scientific data, i.e. mainly *in-vitro* or *in-vivo* experimental research and simulations of exposure in humans. Information regarding the health impact of regular and cumulative intake of food additives in humans and the potential ‘cocktail’ effects/interactions of mixtures is still missing.

Recently, evidence has accumulated suggesting an association between the consumption of “ultra-processed foods”^3^ (which generally contain a wide range of food additives) and increased risk of several chronic diseases, with more than 25 epidemiological studies published worldwide^4–6^, including several from the NutriNet-Santé cohort^7–13^. Beyond a poorer nutritional quality on average^7,14–17^, potential presence of neo-formed compounds and substances migrating from packaging, one of the main hypotheses postulated to explain these results is the presence of food additives^18–27^. New research is needed to better understand health impacts of food processing, formulation and packaging, to be able to provide consumers with safer and more sustainable food products in the coming years^28,29^.

Previous study conducted by this group investigated the distribution and co-occurrence of food additives in a large-scale database of food products available on the French market^30^. Food additives were widespread with more than 50% of industrial food products containing at least 1 and 11.3% at least 5 additives. Most additives probably have no detrimental impact on health (some may even have beneficial effects: e.g. anti-microbial, antioxidants, polyphenols), however, some concerning results, mainly derived from animal and/or cell-based experimental studies, have recently emerged regarding several additives. For instance, nitrates/nitrites^24–26^, carrageenans^31^, glutamate^32–34^, bixin^35,36^, artificial sweeteners^21,37–40^, phosphates^41,42^, emulsifiers^43–46^, caramel^47^, tartrazine^48,49^ and butylated hydroxyanisole/ butylated hydroxytoluene (BHA/BHT)^48^ were previously linked to metabolic, gut microbiota or endocrine perturbations along with carcinogenic, inflammatory and/or oxidative stress effects. Also, in May 2021, following an updated evaluation, EFSA reviewed its position on titanium dioxide (TiO_2_) and stated that this additive could no longer be considered as safe^22,50–52^. Besides, some experimental results suggest potential interactions between some food additives and/or with the food matrix leading to synergistic or antagonist effects on health 4, yet profiles of exposure to additive mixtures in humans are poorly documented.

As a prerequisite for future etiological studies, the present work aimed (1) to estimate the average daily intake of a wide range of food additives in French adults, using detailed data collected in the NutriNet-Santé cohort and (2) to identify the main mixtures of food additives consumed and describe the corresponding profiles of consumers in terms of socio-demographic characteristics, food additive intake and food consumption. The novelty of this work lies in the fact that, to our knowledge, no large-scale population-based study previously estimated the exposure to such a wide variety of food additives with this degree of accuracy, identifying the main mixtures consumed.

## Methods

### Study population

NutriNet-Santé is a French ongoing web-based cohort launched in 2009, which aims to study the associations between nutrition and health as well as the determinants of eating behaviors and nutritional status. It was previously described in detail^53^. Briefly, the only eligibility criteria is to be aged 18 years or older and to have access to the internet. Various means are used for the recruitment of participants from the general population. First, large and repeated multimedia campaigns (television, radio, national and regional newspapers, posters, and Internet) disseminate information about the study and its website. The call for participation is also posted regularly on various websites (national health institutions, city councils, private firms) and regularly relayed by professional channels (e.g., general practitioners and medical specialists, pharmacists, dentists, municipalities)^54^. Recruitment is still open. Participants are tracked using an online platform connected to their email address and questionnaires are completed online on a dedicated website (https://etude-nutrinet-sante.fr). All participants provide an electronic informed consent. The NutriNet-Santé study is registered on ClinicalTrials.gov as NCT03335644 and is conducted according to the Declaration of Helsinki guidelines and is approved by the Institutional Review Board of the French Institute for Health and Medical Research (IRB Inserm) and the “Commission Nationale de l’Informatique et des Libertés” (CNIL n°908,450/n°909,216).

### Data collection

At inclusion and at least each year thereafter, participants were asked to complete a set of five questionnaires related to sociodemographic and lifestyle characteristics (e.g., date of birth, sex, educational level, smoking status)^55^, anthropometry^56,57^, physical activity (validated seven day International Physical Activity Questionnaire, IPAQ)^58^, health status, and dietary intakes.

### Dietary assessment

At inclusion, and every six months thereafter (to vary the season of completion) participants completed a series of three non-consecutive, web-based 24 h-dietary records (validated against an interview by a trained dietitian^59^ and against blood and urinary biomarkers^60,61^), randomly assigned over a two-week period (two week-days and one weekend day). Participants reported all foods and beverages consumed for the three main meals and on any additional eating occasions. Portion sizes were estimated by participants using validated photographs or usual serving containers^62^. To assess daily intakes of macronutrients, micronutrients, alcohol, and total calories, dietary consumption data were linked to the NutriNet-Santé food composition database which contains more than 3,500 generic items^63^. Besides, for each industrial product, the brand and commercial name were collected. Intakes from composite dishes were estimated by referring to French recipes as defined by nutrition professionals. Baseline habitual dietary intakes were averaged from all 24 h-dietary records provided during the first two years of follow-up (at least two 24 h records mandatory for inclusion in the study). Identification of dietary under-reporting was identified based on the method proposed by Black, by using the basal metabolic rate and Goldberg cut-off, and under-reporters of energy intake were excluded^64^.

### Food additives

The determination of food additive exposure is described in detail in Appendix 1. Briefly, for each food (or beverage, candy, chewing-gum) consumed, the presence of each food additive (qualitative composition data) and if relevant, its dose (quantitative composition data) were searched by the research team (no specific knowledge on food additives or their classification was required from the participants). To determine the qualitative presence/absence of food additives, three databases were used: OQALI^65^, a national database hosted by the French food safety agency (ANSES) to characterize the quality of the food supply, Open Food Facts, an open collaborative database of food products marketed worldwide^66^, and Mintel GNPD^67^, an online database of innovative food products in the world. When several composition data existed for a same product at different dates (reformulations), the date of consumption in the cohort (year) was considered in the matching of composition data (dynamic matching).

The quantitative composition of additives has been derived from several sources. Firstly, 2677 ad-hoc laboratory assays were carried out for the main additive-vector food pairs (ad hoc assays commended by our laboratory or by the consumers’ association "UFC Que Choisir"), prioritizing the most consumed additives and those with suspected health effects. The second choice was the use of doses by food categories transmitted by EFSA. Last, when no dose was available neither from assays nor from EFSA, doses from the Codex General Standard for Food Additives (GSFA)^2^ were used. The decision tree in Appendix 1 describes this process in detail. Despite this multi-source assessment, no dose data was available for some additive-vector food pairs (however, it mostly concerned some additives that were consumed by less than 10% of the participants). Thus, only additives for which > 80% of the declarations could be matched to a dose were retained for analysis (i.e. 90 additives). For these, missing doses were imputed by taking the average dose in all food products containing the specific additive.

### Statistical analyses

Descriptive analyses were weighted according to the 2016 French national census report data by using the CALMAR macro run by sex and based on categories for age, socio-professional status and housing area^68^. Intakes of each food additive were described in mg per day as well as in mg/kg bodyweight per day (% of consumers, mean, SD, median, 95^th^ percentile). Toxicity of each food additive is assessed by EFSA to determine its Acceptable Daily Intake (ADI), which is then used to set maximum authorized levels in the different food groups. However, additives without a specified ADI can be used *quantum satis*, i.e., with ‘no limitations other than current good manufacturing practice'. Proportion of participants exceeding the ADI^69^ (when available) were calculated for each food additive. To evaluate the variation that may have been caused by reformulations across a 10y period, the top 50 most consumed food additives was compared between 3 different periods in a sensitivity analysis (2009–2013/2013–2017/2017–2020).

Nonnegative Matrix Factorization (NMF) was used to determine food additive profiles of exposure. This size reduction technique is specifically adapted to sparse matrixes containing positive values^70^. It is described in detail in Appendix 2. Choice of algorithm was carried out according to the measure of residuals and of sparseness^71^, and the number of ranks r was determined according to the method proposed by Brunet et al^72^, using the smallest value of r for which this coefficient starts decreasing. The NMF was performed using the R package NMF^73^. Then, the scores arising from the components were scaled and introduced to a k-means clustering process, and the number of clusters of participants was determined using the elbow method, which examines the percentage of variance explained depending on the number of clusters.

Clusters of participants were described in terms of socio-demographic characteristics, food additive intake and food consumption. Regarding food and food additive consumption, means adjusted for energy intake and number of dietary records were used for description. Comparisons between clusters were performed by Chi-square tests or linear regression, as appropriate.

Partial correlation corresponds to the degree of association between two variables, controlling for other variables. To visualize the partial correlations between food additives, a network was generated using the *glasso* package^74^ which computes a sparse gaussian graphical model with graphical lasso^75^. It can be interpreted as follows: when two food additives are connected by a blue line, it means that they are more consumed by the same participants, when they are connected by a red line, it means that they are rarely consumed by the same participants. Bolder is the line, higher is the correlation. The network was generated for the 90 analyzed food additives.

R version 3.6.2 (R Foundation, Vienna, Austria) was used for the analyses.

## Results

### Most consumed food additives

**Figure 1:**
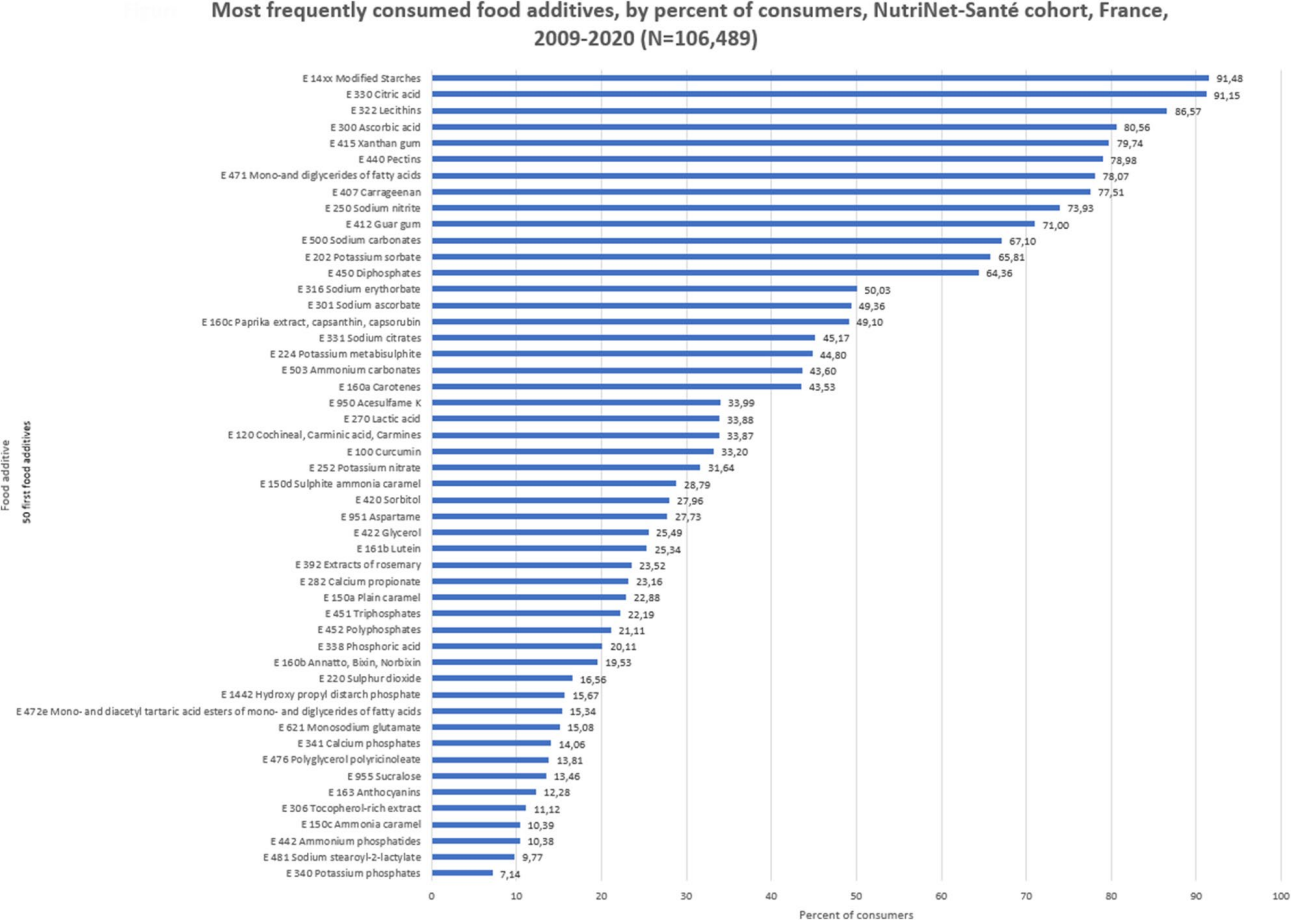
Most frequently consumed food additives, by percent of consumers, NutriNet-Santé cohort, France, 2009–2020 (N = 106,489).

Up to 132,222 participants had at least two 24-h dietary records during the first two years of follow-up. After exclusion of under-reporters, 106,489 participants (69% women) were included for analyses (flowchart is in Appendix 3). Mean age at baseline was 42.9 years (SD = 16.1), and mean number of dietary records was 5.6 (SD = 3.1). Table 1 displays mean and median intakes, by percentage of consumers for each additive. Figure 1 illustrates the 50 most frequently consumed food additives, by percent of consumers. Forty-eight additives were consumed by more than 10% of the participants, with modified starches and citric acid consumed by more than 91.1%. The top 50 also included several food additives for which adverse health effects have been suggested by experimental studies: lecithins (86.6% consumers), mono- and diglycerides of fatty acids (78.1%), carrageenan (77.5%), sodium nitrite (73.9%), di-, tri- and polyphosphates (70.1%), potassium sorbate (65.8%), potassium metabisulphite (44.8%), acesulfame K (34.0%), cochineal (33.9%), potassium nitrate (31.6%), sulfite ammonia caramel (28.8%), bixin (19.5%), monosodium glutamate (15.1%) and sucralose (13.5%). Very little changes were observed in the top 50 generated across three different periods (2009–2013, 2013–2017, 2017–2020) (Appendix 4). Appendix 5 describes the main contributors to each food additive intake in term of number of declarations. In general, the proportion of participants exceeding the ADI was limited, but higher when considering consumers only, with highest proportions being observed for monosodium glutamate (7.07% among all participants, 46.74% among consumers of this specific additive), extracts of rosemary (0.32% overall, 1.36% among consumers), iron oxides and hydroxides (0.23% overall, 4.40% among consumers), sodium metabisulphite (0.18% overall, 4.35% among consumers), potassium sorbate (0.15% overall, 0.23% among consumers), beta-apo-8'-carotenal (0.13% overall, 6.95% among consumers), potassium metabisulphite (0.11% overall, 0.24% among consumers) and calcium phosphates (0.10% overall, 0.71% among consumers).

**Table 1:** Description of intakes of the 90 selected food additives, NutriNet-Santé cohort, France 2009–2020 (N = 106,489).

Food additive	% of consumers	Median (mg/day)	95th percentile (mg/day)	Mean (SD) (mg/day)	Median (mg/kg bw/day)	95th percentile (mg/kg bw/day)	Mean (SD) (mg/kg bw/day)	ADI (mg/kg bw/day)	% of participants exceeding the ADI (overall population)	% of participants exceeding the ADI (among consumers only)
E 14xx Modified Starches	91.48	1304.66	4270.38	1596.45 (1394.44)	19.81	66.42	24.33 (21.84)	NA	NA	NA
E 330 Citric acid	91.15	1380.84	6193.84	1956.51 (2231.41)	20.71	95.19	29.84 (34.31)	NA	NA	NA
E 322 Lecithins	86.57	35.36	168.33	53.94 (71.99)	0.53	2.64	0.83 (1.1)	NA	NA	NA
E 300 Ascorbic acid	80.56	6.33	62.95	15.85 (32.85)	0.10	0.99	0.25 (0.53)	NA	NA	NA
E 415 Xanthan gum	79.74	303.60	1401.31	439.26 (508.65)	4.63	21.08	6.62 (7.62)	NA	NA	NA
E 440 Pectins	78.98	111.49	697.01	199.74 (301.9)	1.71	10.62	3.04 (4.63)	NA	NA	NA
E 471 Mono-and diglycerides of fatty acids	78.07	101.90	547.24	160.8 (197.42)	1.54	8.39	2.44 (2.98)	NA	NA	NA
E 407 Carrageenan	77.51	18.96	179.01	46.55 (69.88)	0.29	2.70	0.71 (1.07)	75	0	0
E 250 Sodium nitrite	73.93	0.12	0.93	0.28 (0.84)	0.00	0.01	0 (0.01)	0.07	0.45	0.61
E 412 Guar gum	71.00	187.45	1070.91	310.41 (399.22)	2.86	16.13	4.66 (5.93)	NA	NA	NA
E 500 Sodium carbonates	67.10	560.00	4950.00	1267.46 (1913.51)	8.51	76.73	19.62 (29.73)	NA	NA	NA
E 202 Potassium sorbate	65.81	6.40	80.51	19.44 (34.03)	0.10	1.22	0.29 (0.5)	3	0.15	0.23
E 450 Diphosphates	64.36	62.86	715.67	178.33 (308.05)	0.95	11.15	2.74 (4.67)	40	0.02	0.03
E 316 Sodium erythorbate	50.03	0.00	32.19	6.67 (14.69)	0.00	0.46	0.1 (0.22)	6	0	0
E 301 Sodium ascorbate	49.36	0.00	29.04	6.57 (12.18)	0.00	0.43	0.1 (0.18)	NA	NA	NA
E 160c Paprika extract, capsanthin, capsorubin	49.10	0.00	0.86	0.17 (0.38)	0.00	0.01	0 (0.01)	24	0	0
E 331 Sodium citrates	45.17	0.00	401.79	84.22 (218.57)	0.00	5.91	1.26 (3.24)	NA	NA	NA
E 224 Potassium metabisulphite	44.80	0.00	8.22	1.48 (5.01)	0.00	0.13	0.02 (0.07)	0.7	0.11	0.24
E 503 Ammonium carbonates	43.60	0.00	2380.00	484.99 (1091.59)	0.00	36.54	7.42 (16.22)	NA	NA	NA
E 160a Carotenes	43.53	0.00	13.47	2.16 (8.71)	0.00	0.21	0.03 (0.13)	NA	NA	NA
E 950 Acesulfame K	33.99	0.00	22.93	4.31 (14.26)	0.00	0.33	0.06 (0.21)	NA	NA	NA
E 270 Lactic acid	33.88	0.00	10.61	1.87 (5.03)	0.00	0.16	0.03 (0.08)	NA	NA	NA
E 120 Cochineal, Carminic acid, Carmines	33.87	0.00	1.74	0.33 (1.33)	0.00	0.03	0.01 (0.02)	5	0	0
E 100 Curcumin	33.20	0.00	6.16	0.94 (3.45)	0.00	0.09	0.01 (0.05)	NA	NA	NA
E 252 Potassium nitrate	31.64	0.00	0.93	0.18 (0.43)	0.00	0.01	0 (0.01)	3.7	0	0
E 150d Sulphite ammonia caramel	28.79	0.00	588.54	100.38 (337.42)	0.00	8.73	1.49 (5.02)	300	0	0
E 420 Sorbitol	27.96	0.00	274.73	48.19 (155.44)	0.00	4.05	0.74 (2.36)	NA	NA	NA
E 951 Aspartame	27.73	0.00	49.92	8.63 (30.58)	0.00	0.72	0.13 (0.47)	40	0	0
E 422 Glycerol	25.49	0.00	809.46	143.92 (509.56)	0.00	12.70	2.24 (7.73)	NA	NA	NA
E 161b Lutein	25.34	0.00	0.71	0.42 (3.44)	0.00	0.01	0.01 (0.05)	1	0.05	0.18
E 392 Extracts of rosemary	23.52	0.00	6.10	0.94 (2.94)	0.00	0.09	0.01 (0.04)	0.3	0.32	1.36
E 282 Calcium propionate	23.16	0.00	57.52	9.89 (29.18)	0.00	0.92	0.15 (0.45)	NA	NA	NA
E 150a Plain caramel	22.88	0.00	47.98	7.54 (32.57)	0.00	0.73	0.11 (0.49)	300	0	0
E 451 Triphosphates	22.19	0.00	274.34	46.88 (150.79)	0.00	4.18	0.69 (2.24)	40	0.02	0.02
E 452 Polyphosphates	21.11	0.00	178.57	36.18 (170.87)	0.00	2.65	0.54 (2.57)	40	0.02	0.03
E 338 Phosphoric acid	20.11	0.00	76.83	12.71 (44.6)	0.00	1.15	0.19 (0.66)	NA	NA	NA
E 160b Annatto, Bixin, Norbixin	19.53	0.00	0.49	0.07 (0.28)	0.00	0.01	0 (0)	0.3	0	0
E 220 Sulphur dioxide	16.56	0.00	0.47	0.14 (1.17)	0.00	0.01	0 (0.02)	0.7	0.01	0.03
E 1442 Hydroxy propyl distarch phosphate	15.67	0.00	386.90	55.7 (194.75)	0.00	5.72	0.85 (3.08)	NA	NA	NA
E 472e Mono- and diacetyl tartaric acid esters of mono- and diglycerides of fatty acids	15.34	0.00	92.86	15.04 (61.38)	0.00	1.44	0.23 (0.95)	NA	NA	NA
E 621 Monosodium glutamate	15.08	0.00	4605.45	2364.55 (20,165.78)	0.00	72.11	36.47 (310.27)	30	7.07	46.74
E 341 Calcium phosphates	14.06	0.00	91.05	23.51 (204.58)	0.00	1.39	0.38 (3.4)	40	0.1	0.71
E 476 Polyglycerol polyricinoleate	13.81	0.00	21.43	3.47 (16.14)	0.00	0.31	0.05 (0.25)	25	0	0
E 955 Sucralose	13.46	0.00	6.50	1.61 (15.1)	0.00	0.10	0.02 (0.24)	NA	NA	NA
E 163 Anthocyanins	12.28	0.00	7.14	1.25 (7.04)	0.00	0.11	0.02 (0.11)	NA	NA	NA
E 306 Tocopherol-rich extract	11.12	0.00	0.45	0.08 (0.39)	0.00	0.01	0 (0.01)	NA	NA	NA
E 150c Ammonia caramel	10.39	0.00	26.79	6.04 (40.08)	0.00	0.41	0.09 (0.62)	300	0	0
E 442 Ammonium phosphatides	10.38	0.00	10.71	4.46 (27.84)	0.00	0.17	0.07 (0.43)	30	0	0
E 481 Sodium stearoyl-2-lactylate	9.77	0.00	30.48	6.01 (32.42)	0.00	0.47	0.09 (0.51)	22	0	0
E 340 Potassium phosphates	7.14	0.00	1.07	2.72 (44.61)	0.00	0.02	0.04 (0.75)	40	0.01	0.09
E 200 Sorbic acid	6.20	0.00	1.97	0.87 (5.5)	0.00	0.03	0.01 (0.09)	3	0	0.06
E 334 Tartaric acid (L( +)-)	5.86	0.00	2.38	1.34 (13.65)	0.00	0.03	0.02 (0.21)	NA	NA	NA
E 133 Brilliant Blue FCF	5.56	0.00	0.00	0.15 (1.67)	0.00	0.00	0 (0.03)	6	0	0
E 172 Iron oxides and hydroxides	5.16	0.00	0.02	0.53 (3.87)	0.00	0.00	0.01 (0.06)	0.5	0.23	4.4
E 385 Calcium disodium ethylene diamine tetra-acetate (Calcium disodium EDTA)	5.07	0.00	0.00	0.02 (0.11)	0.00	0.00	0 (0)	NA	NA	NA
E 339 Sodium phosphates	4.80	0.00	0.00	3.17 (25.42)	0.00	0.00	0.05 (0.4)	40	0	0.02
E 475 Polyglycerol esters of fatty acids	4.26	0.00	0.00	7.3 (55.9)	0.00	0.00	0.11 (0.88)	NA	NA	NA
E 223 Sodium metabisulphite	4.17	0.00	0.00	0.42 (4.08)	0.00	0.00	0.01 (0.06)	0.7	0.18	4.35
E 211 Sodium benzoate	4.02	0.00	0.00	0.4 (5.69)	0.00	0.00	0.01 (0.1)	5	0	0.05
E 960 Steviol glycosides	3.48	0.00	0.00	0.09 (1.44)	0.00	0.00	0 (0.02)	NA	NA	NA
E 102 Tartrazine	3.23	0.00	0.00	0.16 (1.28)	0.00	0.00	0 (0.02)	NA	NA	NA
E 131 Patent Blue V	3.16	0.00	0.00	0.01 (0.34)	0.00	0.00	0 (0.01)	NA	NA	NA
E 473 Sucrose esters of fatty acids	3.15	0.00	0.00	1.12 (11.27)	0.00	0.00	0.02 (0.18)	40	0	0
E 150 Caramel	2.93	0.00	0.00	39.03 (670.33)	0.00	0.00	0.58 (9.48)	300	0.01	0.42
E 234 Nisin	2.24	0.00	0.00	0 (0.01)	0.00	0.00	0 (0)	1	0	0
E 954 Saccharins	2.11	0.00	0.00	0.43 (8)	0.00	0.00	0.01 (0.13)	NA	NA	NA
E 160e Beta-apo-8'-carotenal (C 30)	1.90	0.00	0.00	0.02 (0.31)	0.00	0.00	0 (0)	0.05	0.13	6.95
E 472 Esters of mono- and diglycerides	1.87	0.00	0.00	5.61 (56.47)	0.00	0.00	0.09 (0.85)	NA	NA	NA
E 304 Fatty acid esters of ascorbic acid	1.86	0.00	0.00	0.19 (3.81)	0.00	0.00	0 (0.06)	NA	NA	NA
E 445 Glycerol esters of wood rosins	1.80	0.00	0.00	0.14 (1.73)	0.00	0.00	0 (0.03)	12.5	0	0
E 110 Sunset Yellow FCF/Orange Yellow S	1.50	0.00	0.00	0.07 (0.75)	0.00	0.00	0 (0.01)	1	0	0.06
E 222 Sodium hydrogen sulphite	1.39	0.00	0.00	0.04 (0.62)	0.00	0.00	0 (0.01)	0.7	0	0.2
E 320 Butylated hydroxyanisole (BHA)	1.12	0.00	0.00	0.09 (1.09)	0.00	0.00	0 (0.02)	NA	NA	NA
E 249 Potassium nitrite	0.96	0.00	0.00	0.03 (0.48)	0.00	0.00	0 (0.01)	NA	NA	NA
E 251 Sodium nitrate	0.85	0.00	0.00	0.01 (0.24)	0.00	0.00	0 (0)	3.7	0	0
E 952 Cyclamates	0.67	0.00	0.00	0.04 (0.98)	0.00	0.00	0 (0.01)	NA	NA	NA
E 321 Butylated hydroxytoluene (BHT)	0.59	0.00	0.00	0 (0.05)	0.00	0.00	0 (0)	0.25	0	0.16
E 212 Potassium benzoate	0.56	0.00	0.00	0.06 (1.2)	0.00	0.00	0 (0.02)	NA	NA	NA
E 104 Quinoline Yellow	0.35	0.00	0.00	0.01 (0.18)	0.00	0.00	0 (0)	NA	NA	NA
E 280 Propionic acid	0.24	0.00	0.00	0.01 (0.33)	0.00	0.00	0 (0.01)	NA	NA	NA
E 302 Calcium ascorbate	0.19	0.00	0.00	0 (0.04)	0.00	0.00	0 (0)	NA	NA	NA
E 444 Sucrose acetate isobutyrate	0.13	0.00	0.00	0.02 (0.75)	0.00	0.00	0 (0.01)	20	0	0
E 482 Calcium stearoyl-2-lactylate	0.12	0.00	0.00	0.05 (2.18)	0.00	0.00	0 (0.04)	22	0	0
E 962 Salt of aspartame-acesulfame	0.07	0.00	0.00	0 (0.05)	0.00	0.00	0 (0)	NA	NA	NA
E 129 Allura Red AC	0.06	0.00	0.00	0 (0.05)	0.00	0.00	0 (0)	7	0	0
E 242 Dimethyl dicarbonate	0.05	0.00	0.00	0.01 (0.66)	0.00	0.00	0 (0.01)	NA	NA	NA
E 132 Indigotine, Indigo carmine	0.05	0.00	0.00	0 (0)	0.00	0.00	0 (0)	5	0	0
E 319 Tertiary-butyl hydroquinone (TBHQ)	0.05	0.00	0.00	0 (0.08)	0.00	0.00	0 (0)	0.7	0	0
E 435 Polyoxyethylene sorbitan monostearate (polysorbate 60)	0.05	0.00	0.00	0.01 (0.95)	0.00	0.00	0 (0.02)	25	0	0
E 285 Sodium tetraborate (borax)	0.03	0.00	0.00	0.01 (0.45)	0.00	0.00	0 (0.01)	0.16	0	15.15

Weighted according to the French national census report data by using the CALMAR macro run by sex and based on age, socio-professional category and housing area.

### Food additive mixtures derived by NMF and clusters of participants according to additive intake

**Figure 2:**
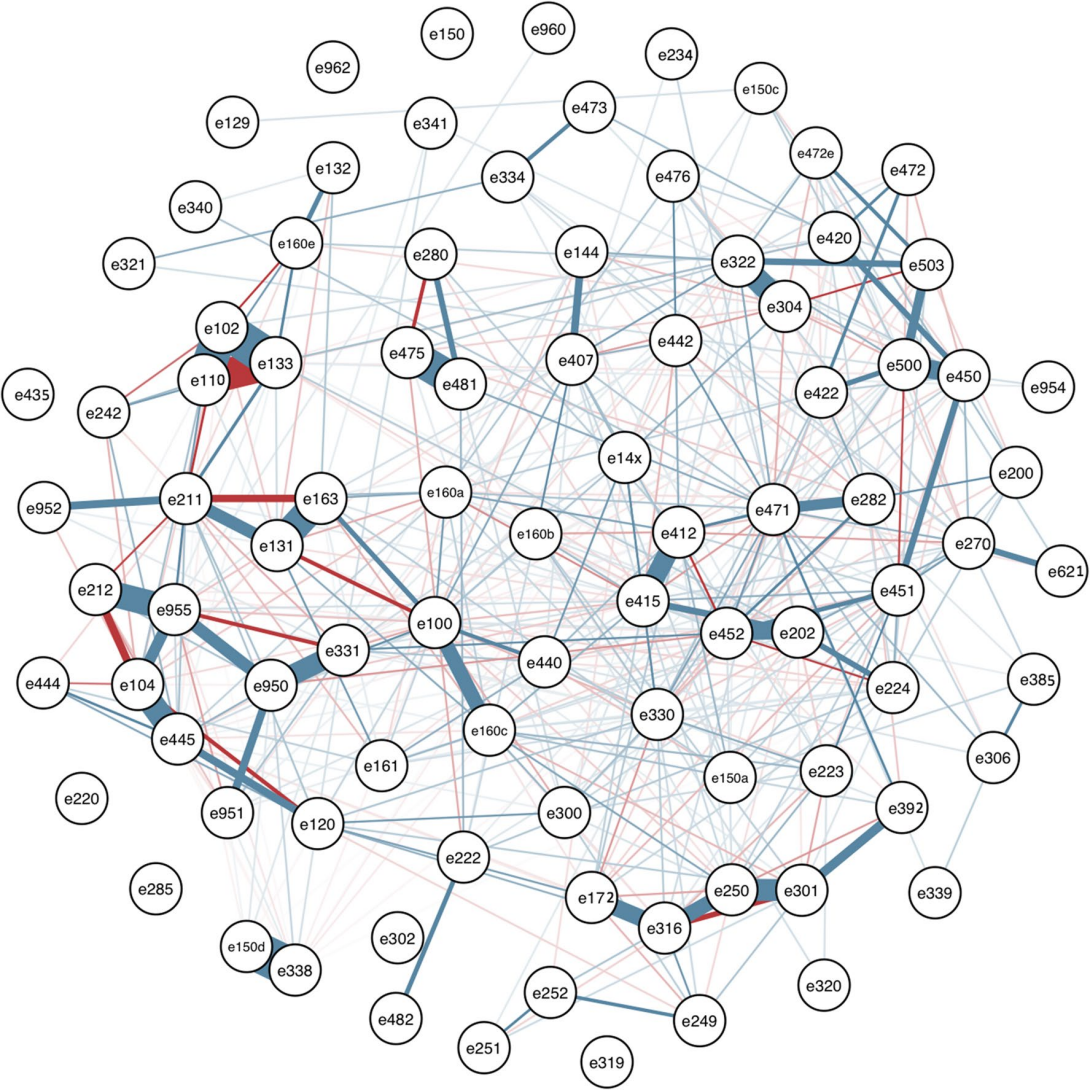
Network of partial correlations between food additive intakes, NutriNet-Santé cohort, France, 2009–2020 (N = 106,489). Graphical lasso. *Glasso* package. Generated for the selected food additives.

The NMF procedure resulted in 5 components that discriminated food additive exposure profiles (Appendix 6a). Figure 2 displays the network of partial correlation of food additives generated with graphical lasso. This method, complementary to NMF, yielded overall consistent results in terms of mixtures of additives identified.

**Figure 3:**
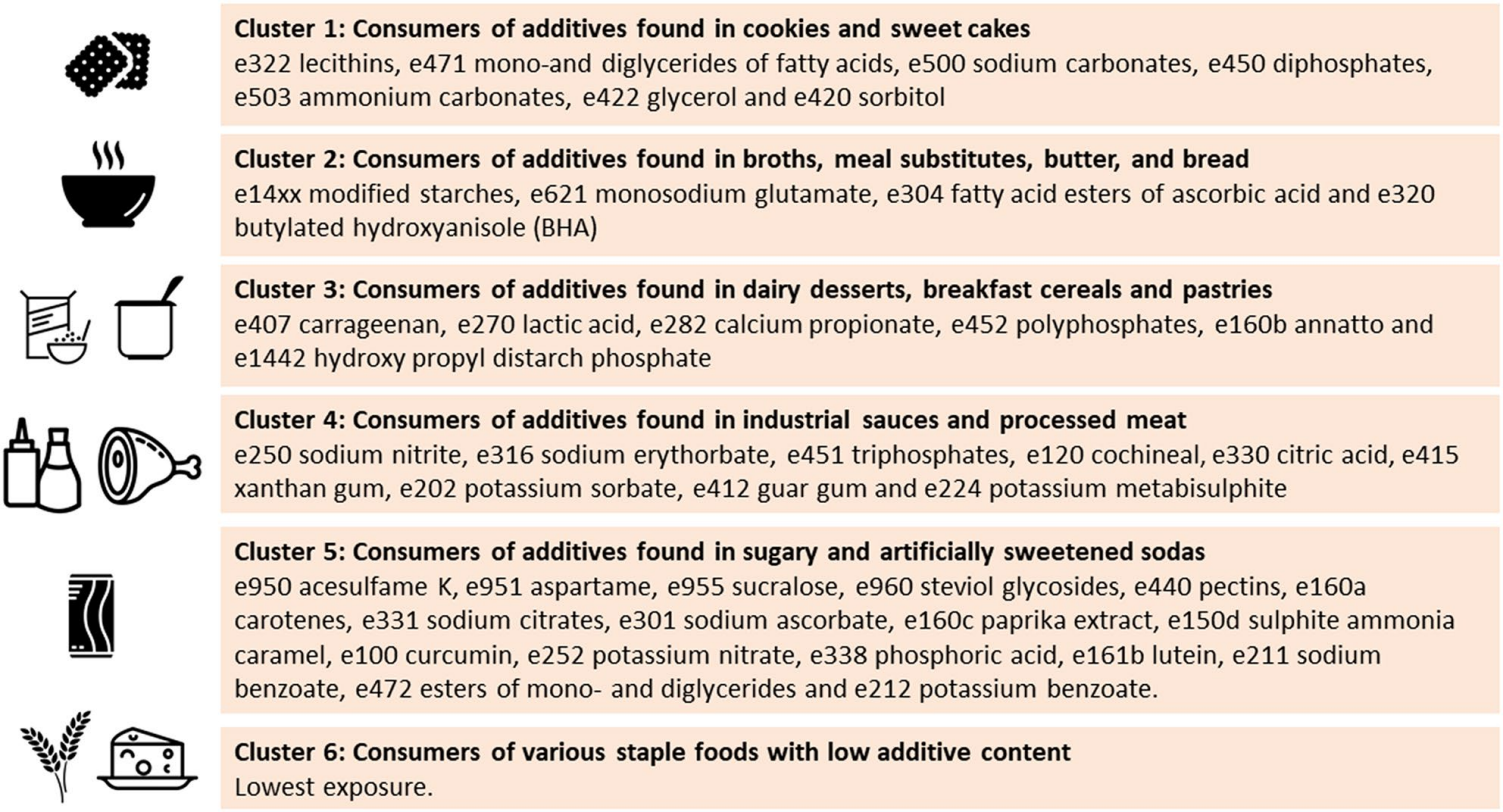
Synthesis of cluster intakes in food groups and additives, NutriNet-Santé cohort, France, 2009–2020 (N = 106,489).

After scaling NMF components of food additive mixtures, k-means clustering was performed and 6 clusters of participants were extracted using the elbow method. Appendix 6b shows the mean of each scaled NMF component, by cluster of participants. Each of the first 5 clusters mostly corresponded to one of the 5 specific food additive mixture NMF component, while cluster 6 corresponded to the participants with an overall low additive exposure. Table 2 displays sociodemographic and lifestyle characteristics according to clusters of participants. Table 3 and 4 display the mean (SD) consumption of food additives and food groups (respectively) by cluster, adjusted for energy intake and number of dietary records. Figure 3 displays a synthesis of cluster’s consumptions of food additives and food groups. The clusters were described as follows:

**Table 2:** Sociodemographic and lifestyle characteristics of clusters of food additive consumers, NutriNet-Santé cohort, France, 2009–2020 (N = 106,489).

Characteristics	All participants (N = 106,489)	Cluster 1 (N = 10,478)	Cluster 2 (N = 15,678)	Cluster 3 (N = 8,944)	Cluster 4 (N = 13,112)	Cluster 5 (N = 2,753)	Cluster 6 (N = 55,524)	*P* value*
Main additive intakes		Consumers of additives found in cookies and sweet cakes	Consumers of additives found in broths, meal substitutes, butter and bread	Consumers of additives found in dairy desserts, breakfast cereals and pastries	Consumers of additives found in industrial sauces and processed meat	Consumers of additives found in sugary and artificially sweetened sodas	Consumers of various staple foods with low additive content	
Percent of the sample	100	9.84	14.72	8.40	12.31	2.59	52.14	
Age in years (mean (SD))	42.94 (16.13)	36.53 (13.6)	48.54 (15.91)	39.59 (15.55)	40.56 (16.14)	34.98 (10.96)	44.06 (15.85)	< 0.001
Women	69.28	63.52	70.61	64.96	55.34	73.02	74.3	< 0.001
Height in cm (mean (SD))	166.7 (8.56)	167.88 (9)	166.28 (8.45)	167.65 (8.54)	168.12 (8.53)	167.71 (8.56)	166.05 (8.36)	< 0.001
Body mass index in kg/m^2^ (mean (SD))	23.73 (4.58)	23.2 (4.32)	24.12 (4.36)	23.98 (4.69)	24.35 (5.14)	25.21 (6)	23.46 (4.37)	< 0.001
IPAQ physical activity level:								
High	30.68	24.70	33.60	32.17	31.21	25.34	30.86	< 0.001
Moderate	35.69	36.78	36.18	32.27	33.32	35.34	36.55	< 0.001
Low	18.71	20.58	17.02	20.56	18.05	23.61	18.48	< 0.001
Education level:								
Primary	4.30	2.63	5.45	3.16	6.98	1.94	3.86	< 0.001
Secondary	37.87	34.57	40.35	41.14	39.36	35.44	36.95	< 0.001
Undergraduate	4.30	25.82	23.23	26.05	21.81	28.05	24.74	< 0.001
Postgraduate	27.29	32.57	23.94	24.99	24.13	29.99	28.36	< 0.001
Smoking status:								
Never	49.57	57.41	51.98	55.16	47.78	42.64	47.15	< 0.001
Former	33.65	25.59	37.4	28.79	31.91	30.05	35.56	< 0.001
Current	16.60	16.87	10.48	15.88	20.19	27.25	17.06	< 0.001
Energy intake without alcohol in kcal/d (mean (SD))	1898.65 (497.27)	2084.59 (542.2)	1891.2 (467.13)	2016.21 (493.93)	2052.22 (524.03)	1914.62 (562.42)	1809.66 (449.18)	< 0.001
Alcohol intake in g/d (mean (SD))**	8.01 (0.04)	5.06 (0.11)	6.76 (0.09)	5.99 (0.12)	9.04 (0.10)	5.44 (0.23)	9.12 (0.05)	< 0.001
Carbohydrate intake in g/d (mean (SD)**	197.50 (0.10)	201.34 (0.34)	202.34 (0.27)	203.44 (0.36)	194.79 (0.29)	197.89 (0.68)	195.04 (0.15)	< 0.001
Lipid intake in g/d (mean (SD))**	81.18 (0.04)	84.57 (0.13)	79.27 (0.11)	80.29 (0.14)	81.44 (0.11)	80.77 (0.27)	81.21 (0.06)	< 0.001
Protein intake in g/d (mean (SD))**	79.08 (0.05)	72.90 (0.15)	80.65 (0.12)	78.76 (0.16)	79.34 (0.13)	83.57 (0.31)	79.55 (0.07)	< 0.001
Sodium intake in mg/d (mean (SD))**	2731.28 (2.05)	2502.93 (6.53)	2891.93 (5.19)	2686.52 (7.04)	2806.67 (5.58)	2806.51 (13.16)	2710.69 (2.88)	< 0.001
Proportion of ultra-processed food in the diet in weight	0.34 (0.00)	0.43 (0.00)	0.32 (0.00)	0.40 (0.00)	0.36 (0.00)	0.45 (0.00)	0.31 (0.00)	< 0.001
Proportion of organic food in the diet in weight	0.21 (0.00)	0.19 (0.00)	0.22 (0.00)	0.16 (0.00)	0.15 (0.00)	0.11 (0.01)	0.23 (0.00)	< 0.001

Weighted according to the French national census report data by using the CALMAR macro run by sex and based on age, socio-professional category and housing area^69^. Values are percentages unless stated otherwise.

IPAQ was available for 91,675 participants, education for 99,725, smoking status for 106,242 and proportion of organic food for 28,075 participants.

*Comparisons between clusters using Chi-square tests or linear regressions, as appropriate.

**Adjusted for energy intake and number of 24 h dietary records.

**Table 3:** Intake of food additives according to clusters of food additive consumers, NutriNet-Santé cohort, France, 2009–2020 (N = 106,489).

Food additive	Mean daily food additive intake in mg/d							
	All participants	Cluster 1 (N = 10,478)	Cluster 2 (N = 15,678)	Cluster 3 (N = 8,944)	Cluster 4 (N = 13,112)	Cluster 5 (N = 2,753)	Cluster 6 (N = 55,524)	*P* value*
E 14xx Modified Starches	1586.10 (4.25)	1421.79 (9.59)	3721.10 (7.63)	1833.86 (10.35)	1858.91 (8.21)	1614.79 (19.34)	865.89 (4.23)	< 0.001
E 330 Citric acid	1928.04 (6.72)	2720.71 (20.82)	2421.20 (16.57)	1895.63 (22.47)	2810.04 (17.82)	2246.35 (42.00)	1394.14 (9.18)	< 0.001
E 322 Lecithins	52.74 (0.21)	109.99 (0.65)	42.54 (0.52)	75.81 (0.71)	52.24 (0.56)	73.16 (1.32)	40.42 (0.29)	< 0.001
E 440 Pectins	198.70 (0.93)	188.15 (2.91)	292.04 (2.31)	234.42 (3.14)	267.19 (2.49)	365.19 (5.86)	141.63 (1.28)	< 0.001
E 300 Ascorbic acid	15.69 (0.10)	12.48 (0.16)	10.80 (0.12)	12.21 (0.17)	13.17 (0.13)	11.47 (0.32)	8.58 (0.07)	< 0.001
E 415 Xanthan gum	432.42 (1.53)	426.87 (3.89)	360.25 (3.10)	523.25 (4.20)	1187.87 (3.33)	459.06 (7.85)	244.42 (1.72)	< 0.001
E 471 Mono-and diglycerides of fatty acids	157.67 (0.59)	229.46 (1.84)	143.12 (1.46)	224.47 (1.99)	215.26 (1.58)	205.29 (3.71)	120.62 (0.81)	< 0.001
E 407 Carrageenan	45.83 (0.21)	36.52 (0.47)	32.52 (0.38)	210.03 (0.51)	40.57 (0.41)	53.27 (0.96)	26.18 (0.21)	< 0.001
E 202 Potassium sorbate	19.12 (0.10)	26.04 (0.32)	13.91 (0.25)	26.59 (0.34)	43.74 (0.27)	24.00 (0.64)	11.75 (0.14)	< 0.001
E 412 Guar gum	305.14 (1.20)	267.82 (3.04)	210.15 (2.42)	340.44 (3.29)	912.31 (2.60)	353.50 (6.14)	176.18 (1.34)	< 0.001
E 250 Sodium nitrite	0.27 (0.00)	0.25 (0.00)	0.20 (0.00)	0.24 (0.00)	0.39 (0.00)	0.30 (0.01)	0.18 (0.00)	< 0.001
E 500 Sodium carbonates	1241.62 (5.75)	4631.22 (14.84)	882.56 (11.81)	1304.14 (16.02)	912.12 (12.70)	1420.19 (29.94)	773.57 (6.55)	< 0.001
E 450 Diphosphates	174.44 (0.93)	530.81 (2.74)	133.99 (2.18)	220.76 (2.96)	164.58 (2.35)	189.05 (5.53)	113.44 (1.21)	< 0.001
E 950 Acesulfame K	4.30 (0.04)	4.32 (0.11)	2.83 (0.09)	4.28 (0.12)	4.71 (0.10)	62.75 (0.23)	2.12 (0.05)	< 0.001
E 160a Carotenes	2.11 (0.03)	3.20 (0.09)	1.78 (0.07)	3.01 (0.09)	2.94 (0.07)	4.14 (0.17)	1.55 (0.04)	< 0.001
E 331 Sodium citrates	83.51 (0.67)	79.45 (1.68)	63.54 (1.34)	98.30 (1.81)	93.97 (1.44)	952.36 (3.39)	45.11 (0.74)	< 0.001
E 951 Aspartame	8.67 (0.09)	6.37 (0.26)	6.73 (0.21)	7.65 (0.28)	8.81 (0.22)	107.29 (0.53)	5.28 (0.12)	< 0.001
E 301 Sodium ascorbate	6.48 (0.04)	7.39 (0.12)	5.84 (0.09)	7.19 (0.13)	9.43 (0.10)	9.61 (0.24)	5.49 (0.05)	< 0.001
E 160c Paprika extract, capsanthin, capsorubin	0.17 (0.00)	0.21 (0.00)	0.18 (0.00)	0.25 (0.00)	0.30 (0.00)	0.37 (0.01)	0.11 (0.00)	< 0.001
E 316 Sodium erythorbate	6.57 (0.04)	6.89 (0.14)	5.91 (0.11)	6.50 (0.15)	12.66 (0.12)	8.69 (0.29)	5.05 (0.06)	< 0.001
E 224 Potassium metabisulphite	1.45 (0.02)	3.14 (0.05)	0.85 (0.04)	1.21 (0.05)	3.56 (0.04)	1.75 (0.10)	0.79 (0.02)	< 0.001
E 503 Ammonium carbonates	473.10 (3.31)	2425.29 (8.55)	232.17 (6.80)	353.66 (9.23)	316.65 (7.32)	477.72 (17.25)	234.71 (3.77)	< 0.001
E 270 Lactic acid	1.85 (0.02)	2.22 (0.05)	1.87 (0.04)	2.78 (0.05)	2.57 (0.04)	1.86 (0.10)	1.43 (0.02)	< 0.001
E 150d Sulphite ammonia caramel	98.74 (1.03)	86.84 (2.29)	32.72 (1.82)	91.00 (2.47)	79.84 (1.96)	1652.56 (4.62)	55.22 (1.01)	< 0.001
E 100 Curcumin	0.95 (0.01)	0.75 (0.03)	1.50 (0.03)	2.43 (0.04)	1.11 (0.03)	2.88 (0.07)	0.45 (0.01)	< 0.001
E 120 Cochineal, Carminic acid, Carmines	0.33 (0.00)	0.43 (0.02)	0.35 (0.01)	0.41 (0.02)	0.81 (0.01)	0.69 (0.03)	0.23 (0.01)	< 0.001
E 252 Potassium nitrate	0.18 (0.00)	0.19 (0.00)	0.14 (0.00)	0.19 (0.00)	0.19 (0.00)	0.21 (0.01)	0.18 (0.00)	< 0.001
E 422 Glycerol	140.84 (1.56)	436.34 (4.92)	110.12 (3.91)	154.75 (5.31)	129.28 (4.21)	188.44 (9.92)	92.59 (2.17)	< 0.001
E 282 Calcium propionate	9.76 (0.09)	12.13 (0.29)	7.00 (0.23)	15.94 (0.31)	13.26 (0.25)	13.32 (0.58)	8.08 (0.13)	< 0.001
E 420 Sorbitol	47.05 (0.47)	108.99 (1.51)	35.11 (1.20)	67.04 (1.63)	46.64 (1.29)	57.72 (3.05)	35.28 (0.67)	< 0.001
E 150a Plain caramel	7.38 (0.10)	9.23 (0.32)	5.22 (0.25)	12.76 (0.34)	15.75 (0.27)	11.28 (0.64)	4.48 (0.14)	< 0.001
E 161b Lutein	0.42 (0.01)	0.58 (0.03)	0.60 (0.03)	0.41 (0.04)	0.67 (0.03)	1.16 (0.07)	0.24 (0.01)	< 0.001
E 338 Phosphoric acid	12.50 (0.14)	10.94 (0.30)	3.71 (0.24)	11.46 (0.33)	9.94 (0.26)	218.30 (0.61)	6.76 (0.13)	< 0.001
E 451 Triphosphates	46.02 (0.46)	64.00 (1.45)	34.01 (1.16)	57.72 (1.57)	120.62 (1.24)	53.56 (2.93)	24.73 (0.64)	< 0.001
E 452 Polyphosphates	35.43 (0.52)	28.19 (1.67)	25.18 (1.33)	96.99 (1.80)	71.68 (1.43)	46.16 (3.36)	20.13 (0.74)	< 0.001
E 476 Polyglycerol polyricinoleate	3.42 (0.05)	6.09 (0.16)	2.80 (0.13)	5.76 (0.17)	3.67 (0.14)	5.60 (0.32)	2.55 (0.07)	< 0.001
E 392 Extracts of rosemary	0.93 (0.01)	1.01 (0.03)	1.19 (0.02)	1.00 (0.03)	1.10 (0.02)	1.36 (0.06)	0.76 (0.01)	< 0.001
E 955 Sucralose	1.59 (0.05)	1.97 (0.15)	1.05 (0.12)	1.96 (0.16)	2.17 (0.13)	10.19 (0.30)	1.08 (0.07)	< 0.001
E 160b Annatto, Bixin, Norbixin	0.07 (0.00)	0.06 (0.00)	0.06 (0.00)	0.24 (0.00)	0.10 (0.00)	0.08 (0.01)	0.04 (0.00)	< 0.001
E 1442 Hydroxy propyl distarch phosphate	55.26 (0.60)	46.84 (1.83)	36.94 (1.46)	252.22 (1.98)	43.09 (1.57)	82.98 (3.69)	32.51 (0.81)	< 0.001
E 220 Sulphur dioxide	0.14 (0.00)	0.21 (0.01)	0.12 (0.01)	0.10 (0.01)	0.11 (0.01)	0.50 (0.03)	0.12 (0.01)	< 0.001
E 472e Mono- and diacetyl tartaric acid esters of mono- and diglycerides of fatty acids	14.88 (0.19)	54.62 (0.59)	8.69 (0.47)	17.16 (0.64)	16.52 (0.51)	17.45 (1.19)	8.29 (0.26)	< 0.001
E 621 Monosodium glutamate	2352.51 (61.96)	2801.03 (199.03)	3710.40 (158.38)	1863.33 (214.81)	2542.92 (170.32)	1649.70 (401.44)	1922.60 ( 87.77)	< 0.001
E 306 Tocopherol-rich extract	0.08 (0.00)	0.07 (0.00)	0.06 (0.00)	0.07 (0.00)	0.15 (0.00)	0.11 (0.01)	0.07 (0.00)	< 0.001
E 341 Calcium phosphates	23.25 (0.63)	47.53 (2.02)	27.05 (1.61)	25.91 (2.18)	15.77 (1.73)	29.44 (4.07)	18.72 (0.89)	< 0.001
E 481 Sodium stearoyl-2-lactylate	5.91 (0.10)	12.00 (0.32)	3.19 (0.25)	10.55 (0.34)	6.76 (0.27)	12.05 (0.64)	4.32 (0.14)	< 0.001
E 150c Ammonia caramel	5.94 (0.12)	9.06 (0.40)	6.60 (0.31)	8.91 (0.43)	5.80 (0.34)	8.43 (0.80)	4.59 (0.17)	< 0.001
E 163 Anthocyanins	1.22 (0.02)	1.49 (0.07)	1.13 (0.06)	2.61 (0.07)	1.59 (0.06)	3.32 (0.14)	0.79 (0.03)	< 0.001
E 385 Calcium disodium ethylene diamine tetra-acetate (Calcium disodium EDTA)	0.02 (0.00)	0.02 (0.00)	0.01 (0.00)	0.02 (0.00)	0.02 (0.00)	0.02 (0.00)	0.01 (0.00)	< 0.001
E 960 Steviol glycosides	0.09 (0.00)	0.09 (0.01)	0.10 (0.01)	0.14 (0.02)	0.10 (0.01)	0.21 (0.03)	0.07 (0.01)	< 0.001
E 340 Potassium phosphates	2.72 (0.14)	3.62 (0.44)	2.07 (0.35)	4.01 (0.48)	2.33 (0.38)	9.11 (0.89)	2.35 (0.19)	< 0.001
E 442 Ammonium phosphatides	4.38 (0.09)	3.86 (0.27)	1.97 (0.22)	13.36 (0.30)	6.10 (0.23)	7.19 (0.55)	3.17 (0.12)	< 0.001
E 334 Tartaric acid (L( +)-)	1.31 (0.04)	2.24 (0.13)	1.28 (0.11)	1.16 (0.15)	1.34 (0.12)	1.82 (0.27)	1.14 (0.06)	< 0.001
E 200 Sorbic acid	0.85 (0.02)	2.11 (0.08)	0.85 (0.06)	1.11 (0.08)	1.28 (0.06)	1.18 (0.15)	0.87 (0.03)	< 0.001
E 223 Sodium metabisulphite	0.41 (0.01)	0.23 (0.04)	0.76 (0.03)	0.35 (0.04)	1.24 (0.03)	0.28 (0.08)	0.15 (0.02)	< 0.001
E 211 Sodium benzoate	0.40 (0.02)	0.32 (0.06)	0.22 (0.04)	0.43 (0.06)	0.38 (0.05)	1.73 (0.11)	0.42 (0.02)	< 0.001
E 133 Brilliant Blue FCF	0.15 (0.01)	0.23 (0.02)	0.10 (0.01)	0.19 (0.02)	0.26 (0.01)	0.38 (0.03)	0.12 (0.01)	< 0.001
E 339 Sodium phosphates	3.15 (0.08)	4.03 (0.25)	4.02 (0.20)	3.46 (0.27)	2.97 (0.21)	5.58 (0.51)	2.60 (0.11)	< 0.001
E 172 Iron oxides and hydroxides	0.52 (0.01)	0.35 (0.04)	0.40 (0.03)	0.34 (0.04)	1.84 (0.03)	0.56 (0.08)	0.27 (0.02)	< 0.001
E 475 Polyglycerol esters of fatty acids	7.16 (0.17)	15.58 (0.55)	3.79 (0.44)	11.83 (0.59)	7.55 (0.47)	19.48 (1.11)	5.16 (0.24)	< 0.001
E 954 Saccharins	0.42 (0.02)	0.16 (0.08)	0.30 (0.06)	0.24 (0.09)	1.23 (0.07)	1.29 (0.16)	0.28 (0.03)	< 0.001
E 131 Patent Blue V	0.01 (0.00)	0.12 (0.01)	0.05 (0.01)	0.08 (0.01)	0.08 (0.01)	0.29 (0.02)	0.07 (0.00)	< 0.001
E 150 Caramel	37.96 (2.06)	22.36 ( 6.62)	30.78 ( 5.27)	45.08 ( 7.15)	50.93 ( 5.67)	57.85 (13.36)	37.66 ( 2.92)	< 0.001
E 473 Sucrose esters of fatty acids	1.11 (0.03)	2.36 (0.11)	0.79 (0.09)	1.24 (0.12)	1.25 (0.10)	1.59 (0.22)	0.89 (0.05)	< 0.001
E 102 Tartrazine	0.16 (0.00)	0.18 (0.01)	0.11 (0.01)	0.18 (0.01)	0.31 (0.01)	0.22 (0.03)	0.12 (0.01)	< 0.001
E 445 Glycerol esters of wood rosins	0.14 (0.01)	0.13 (0.02)	0.10 (0.01)	0.20 (0.02)	0.30 (0.01)	0.84 (0.03)	0.08 (0.01)	< 0.001
E 234 Nisin	0.00 (0.00)	0.00 (0.00)	0.00 (0.00)	0.00 (0.00)	0.00 (0.00)	0.00 (0.00)	0.00 (0.00)	< 0.001
E 304 Fatty acid esters of ascorbic acid	0.19 (0.01)	0.17 (0.04)	0.71 (0.03)	0.19 (0.04)	0.11 (0.03)	0.35 (0.08)	0.06 (0.02)	< 0.001
E 160e Beta-apo-8'-carotenal (C 30)	0.02 (0.00)	0.03 (0.00)	0.01 (0.00)	0.02 (0.00)	0.02 (0.00)	0.06 (0.01)	0.01 (0.00)	< 0.001
E 952 Cyclamates	0.04 (0.00)	0.04 (0.01)	0.05 (0.01)	0.06 (0.01)	0.03 (0.01)	0.21 (0.02)	0.03 (0.00)	< 0.001
E 472 Esters of mono- and diglycerides	5.42 (0.17)	5.28 (0.56)	5.53 (0.44)	4.50 (0.60)	5.93 (0.48)	11.72 (1.12)	5.14 (0.25)	< 0.001
E 320 Butylated hydroxyanisole (BHA)	0.09 (0.00)	0.07 (0.01)	0.31 (0.01)	0.06 (0.01)	0.05 (0.01)	0.07 (0.02)	0.05 (0.00)	< 0.001
E 222 Sodium hydrogen sulphite	0.04 (0.00)	0.07 (0.01)	0.03 (0.00)	0.04 (0.01)	0.08 (0.01)	0.06 (0.01)	0.02 (0.00)	< 0.001
E 110 Sunset Yellow FCF/Orange Yellow S	0.07 (0.00)	0.05 (0.01)	0.06 (0.01)	0.08 (0.01)	0.16 (0.01)	0.04 (0.01)	0.05 (0.00)	< 0.001
E 251 Sodium nitrate	0.01 (0.00)	0.01 (0.00)	0.00 (0.00)	0.00 (0.00)	0.00 (0.00)	0.00 (0.00)	0.00 (0.00)	< 0.001
E 212 Potassium benzoate	0.06 (0.00)	0.06 (0.01)	0.04 (0.01)	0.07 (0.01)	0.06 (0.01)	0.37 (0.02)	0.05 (0.01)	< 0.001
E 249 Potassium nitrite	0.03 (0.00)	0.06 (0.00)	0.02 (0.00)	0.03 (0.01)	0.04 (0.00)	0.03 (0.01)	0.02 (0.00)	< 0.001
E 321 Butylated hydroxytoluene (BHT)	0.00 (0.00)	0.00 (0.00)	0.00 (0.00)	0.00 (0.00)	0.00 (0.00)	0.00 (0.00)	0.00 (0.00)	< 0.001
E 104 Quinoline Yellow	0.01 (0.00)	0.01 (0.00)	0.00 (0.00)	0.01 (0.00)	0.00 (0.00)	0.04 (0.00)	0.00 (0.00)	< 0.001
E 280 Propionic acid	0.01 (0.00)	0.16 (0.03)	0.06 (0.03)	0.12 (0.04)	0.37 (0.03)	0.28 (0.07)	0.04 (0.01)	< 0.001
E 482 Calcium stearoyl-2-lactylate	0.05 (0.01)	0.13 (0.02)	0.02 (0.02)	0.12 (0.02)	0.07 (0.02)	0.11 (0.04)	0.02 (0.01)	< 0.001
E 302 Calcium ascorbate	0.00 (0.00)	0.00 (0.00)	0.00 (0.00)	0.00 (0.00)	0.00 (0.00)	0.00 (0.00)	0.00 (0.00)	< 0.001
E 444 Sucrose acetate isobutyrate	0.02 (0.00)	0.03 (0.01)	0.02 (0.01)	0.03 (0.01)	0.03 (0.01)	0.04 (0.02)	0.01 (0.00)	< 0.001
E 242 Dimethyl dicarbonate	0.01 (0.00)	0.00 (0.01)	0.02 (0.01)	0.01 (0.01)	0.01 (0.01)	0.00 (0.01)	0.01 (0.00)	< 0.001
E 129 Allura Red AC	0.00 (0.00)	0.00 (0.00)	0.00 (0.00)	0.00 (0.00)	0.00 (0.00)	0.00 (0.00)	0.00 (0.00)	< 0.001
E 962 Salt of aspartame-acesulfame	0.00 (0.00)	0.00 (0.00)	0.00 (0.00)	0.00 (0.00)	0.00 (0.00)	0.00 (0.00)	0.00 (0.00)	< 0.001
E 132 Indigotine, Indigo carmine	0.00 (0.00)	0.00 (0.00)	0.00 (0.00)	0.00 (0.00)	0.00 (0.00)	0.00 (0.00)	0.00 (0.00)	0.02
E 319 Tertiary-butyl hydroquinone (TBHQ)	0.00 (0.00)	0.01 (0.00)	0.00 (0.00)	0.00 (0.00)	0.00 (0.00)	0.00 (0.00)	0.00 (0.00)	< 0.001
E 285 Sodium tetraborate (borax)	0.01 (0.00)	0.00 (0.00)	0.01 (0.00)	0.00 (0.00)	0.00 (0.00)	0.01 (0.01)	0.01 (0.00)	0.03
E 435 Polyoxyethylene sorbitan monostearate (polysorbate 60)	0.01 (0.00)	0.02 (0.01)	0.01 (0.01)	0.02 (0.01)	0.01 (0.01)	0.02 (0.02)	0.01 (0.00)	< 0.001

Weighted according to the French national census report data by using the CALMAR macro run by sex and based on age, socio-professional category and housing area^68^.

Mean (SD), adjusted for energy intake and number of 24 h dietary records.

*P values for comparisons between clusters using linear regression.

**Table 4:** Consumption of food groups according to clusters of food additive consumers, NutriNet-Santé cohort, France, 2009–2020 (N = 106,489).

Food group	Mean daily food intake in mg/d							
	All participants	Cluster 1 (N = 10,478)	Cluster 2 (N = 15,678)	Cluster 3 (N = 8,944)	Cluster 4 (N = 13,112)	Cluster 5 (N = 2,753)	Cluster 6 (N = 55,524)	*p* value*
Fruits	190.83 (0.45)	159.61 (1.44)	212.19 (1.14)	163.54 (1.58)	166.03 (1.24)	149.41 (2.91)	202.84 (0.64)	< 0.001
Dried fruits	2.29 (0.02)	1.41 (0.08)	2.90 (0.06)	1.53 (0.09)	1.30 (0.07)	1.25 (0.16)	2.69 (0.04)	< 0.001
Salted oleaginous fruits	1.37 (0.01)	1.29 (0.04)	1.04 (0.03)	1.05 (0.05)	1.45 (0.04)	1.55 (0.08)	1.51 (0.02)	< 0.001
Natural oleaginous fruits	3.33 (0.03)	1.50 (0.10)	4.25 (0.08)	1.57 (0.11)	1.55 (0.08)	1.63 (0.19)	4.22 (0.04)	< 0.001
Vegetables	220.02 (0.38)	169.30 (1.19)	274.48 (0.94)	223.02 (1.30)	200.38 (1.02)	188.81 (2.39)	219.16 (0.52)	< 0.001
Pulses	12.09 (0.08)	9.34 (0.24)	12.87 (0.19)	9.85 (0.26)	8.57 (0.21)	7.99 (0.49)	13.82 (0.11)	< 0.001
Meat	43.37 (0.12)	44.81 (0.40)	41.79 (0.32)	45.25 (0.44)	40.96 (0.34)	47.58 (0.80)	43.70 (0.18)	< 0.001
Poultry	25.50 (0.09)	22.97 (0.30)	24.44 (0.24)	27.13 (0.33)	28.65 (0.26)	32.87 (0.60)	24.90 (0.13)	< 0.001
Eggs	14.29 (0.06)	12.72 (0.21)	17.21 (0.16)	11.94 (0.22)	13.76 (0.18)	15.23 (0.41)	14.17 (0.09)	< 0.001
Pork and poultry hams	11.85 (0.05)	12.21 (0.17)	11.01 (0.14)	13.67 (0.19)	13.93 (0.15)	19.76 (0.34)	10.85 (0.08)	< 0.001
Fish and seafood delicatessens	2.81 (0.03)	2.53 (0.10)	2.56 (0.08)	3.07 (0.11)	3.07 (0.08)	6.44 (0.19)	2.66 (0.04)	< 0.001
Fish	30.66 (0.11)	23.06 (0.34)	31.20 (0.27)	26.53 (0.37)	35.93 (0.29)	26.47 (0.68)	31.43 (0.15)	< 0.001
Seafood	7.48 (0.05)	5.87 (0.17)	7.06 (0.13)	6.74 (0.18)	8.12 (0.14)	7.69 (0.33)	7.86 (0.07)	< 0.001
Offal	3.71 (0.04)	2.18 (0.11)	4.30 (0.09)	2.47 (0.13)	2.88 (0.10)	2.65 (0.23)	4.27 (0.05)	< 0.001
Cheeses	36.42 (0.08)	35.03 (0.26)	35.56 (0.20)	33.43 (0.28)	34.22 (0.22)	36.03 (0.52)	38.00 (0.11)	< 0.001
Butter	7.01 (0.02)	5.35 (0.08)	8.29 (0.06)	6.35 (0.08)	6.08 (0.07)	5.39 (0.16)	7.36 (0.03)	< 0.001
Margarines	2.19 (0.02)	1.46 (0.05)	2.74 (0.04)	2.37 (0.06)	2.50 (0.04)	1.34 (0.10)	2.08 (0.02)	< 0.001
Oils	9.34 (0.03)	8.90 (0.08)	8.35 (0.07)	7.46 (0.09)	8.35 (0.07)	8.46 (0.17)	10.31 (0.04)	< 0.001
Other fats	7.36 (0.03)	6.87 (0.09)	12.50 (0.07)	8.15 (0.10)	6.51 (0.08)	6.40 (0.18)	6.04 (0.04)	< 0.001
Pasta	35.45 (0.14)	34.89 (0.44)	31.81 (0.35)	36.26 (0.48)	35.07 (0.38)	38.71 (0.88)	36.49 (0.19)	< 0.001
Potatoes	48.05 (0.14)	42.39 (0.45)	64.31 (0.36)	42.46 (0.49)	47.96 (0.39)	42.63 (0.91)	45.36 (0.20)	< 0.001
Rice	19.37 (0.09)	16.86 (0.30)	16.30 (0.24)	18.09 (0.33)	22.45 (0.26)	18.29 (0.60)	20.24 (0.13)	< 0.001
Rice, wholegrain foods	2.47 (0.04)	1.59 (0.12)	2.89 (0.10)	1.55 (0.14)	1.10 (0.11)	1.31 (0.25)	3.06 (0.05)	< 0.001
Pasta, whole foods	3.04 (0.05)	2.05 (0.15)	2.89 (0.12)	2.24 (0.16)	1.74 (0.13)	2.40 (0.30)	3.75 (0.06)	< 0.001
Semolina	5.23 (0.04)	4.42 (0.14)	4.45 (0.11)	4.62 (0.15)	7.52 (0.12)	4.67 (0.28)	5.16 (0.06)	< 0.001
Other whole grains	0.64 (0.01)	0.55 (0.04)	0.59 (0.03)	0.58 (0.04)	0.38 (0.03)	0.85 (0.08)	0.73 (0.02)	< 0.001
Other starches and tubers	1.27 (0.03)	0.98 (0.09)	1.62 (0.07)	1.00 (0.10)	0.61 (0.08)	0.53 (0.18)	1.46 (0.04)	< 0.001
Breads, rusks, wholemeal foods	25.50 (0.12)	16.74 (0.37)	31.32 (0.29)	19.67 (0.40)	19.67 (0.32)	19.97 (0.74)	28.07 (0.16)	< 0.001
Other cereals	6.72 (0.05)	6.13 (0.17)	6.69 (0.14)	5.48 (0.19)	4.70 (0.15)	4.64 (0.35)	7.64 (0.08)	< 0.001
Flours	0.81 (0.01)	0.61 (0.03)	1.25 (0.02)	0.94 (0.03)	0.49 (0.03)	0.69 (0.06)	0.79 (0.01)	< 0.001
Flours, wholemeal feeds	0.09 (0.00)	0.04 (0.01)	0.10 (0.01)	0.04 (0.01)	0.01 (0.01)	0.03 (0.03)	0.13 (0.01)	< 0.001
Bread, rusks	80.01 (0.18)	68.06 (0.57)	83.50 (0.45)	80.72 (0.62)	86.68 (0.49)	68.50 (1.14)	79.95 (0.25)	< 0.001
Non-alcoholic, unsweetened drinks (except juices)	1057.87 (1.64)	1051.93 (5.22)	1068.47 (4.14)	1034.49 (5.72)	985.89 (4.50)	1265.51 (10.53)	1068.21 (2.31)	< 0.001
Milk	81.46 (0.37)	73.15 (1.19)	86.61 (0.95)	83.96 (1.31)	82.07 (1.03)	82.93 (2.40)	80.87 (0.53)	< 0.001
100% pure fruit juice	52.85 (0.24)	60.91 (0.78)	44.50 (0.62)	62.71 (0.85)	47.23 (0.67)	49.10 (1.57)	53.91 (0.34)	< 0.001
Broths, preparation liquids…	29.15 (0.14)	19.94 (0.39)	78.43 (0.31)	20.76 (0.43)	24.39 (0.34)	15.72 (0.78)	19.12 (0.17)	< 0.001
Alcoholic Beverages	96.39 (0.45)	63.56 (1.42)	79.48 (1.13)	72.92 (1.56)	104.62 (1.22)	67.18 (2.87)	110.69 (0.63)	< 0.001
Sweetened non-alcoholic drinks	48.33 (0.34)	71.18 (1.08)	26.32 (0.85)	54.33 (1.18)	61.09 (0.93)	164.20 (2.17)	41.08 (0.48)	< 0.001
Vegetable Juice	1.64 (0.04)	1.38 (0.14)	1.22 (0.11)	1.50 (0.16)	1.21 (0.12)	1.69 (0.29)	1.95 (0.06)	< 0.001
Fatty and sweet cakes	12.38 (0.07)	24.05 (0.23)	11.46 (0.18)	12.68 (0.25)	12.88 (0.20)	12.71 (0.46)	10.26 (0.10)	< 0.001
Fatty and salty products	6.72 (0.04)	9.19 (0.11)	6.61 (0.09)	7.25 (0.12)	7.01 (0.10)	7.66 (0.23)	6.08 (0.05)	< 0.001
Fatty and sweet cookies	8.26 (0.05)	26.29 (0.13)	6.16 (0.11)	7.57 (0.15)	6.23 (0.11)	8.94 (0.27)	6.06 (0.06)	< 0.001
Sweet products	21.20 (0.06)	17.02 (0.20)	23.54 (0.16)	21.73 (0.22)	20.41 (0.17)	16.00 (0.40)	21.65 (0.09)	< 0.001
Fatty cakes, or sweet cakes, or low-fat and low-sweet cakes	17.72 (0.09)	20.89 (0.27)	17.80 (0.22)	21.02 (0.30)	23.04 (0.24)	16.08 (0.55)	15.30 (0.12)	< 0.001
Viennese pastries	9.27 (0.06)	12.35 (0.18)	6.91 (0.15)	12.96 (0.20)	9.52 (0.16)	13.10 (0.37)	8.58 (0.08)	< 0.001
Aperitif products	4.05 (0.03)	4.74 (0.09)	2.99 (0.07)	3.78 (0.10)	4.52 (0.08)	6.01 (0.19)	4.06 (0.04)	< 0.001
Sweet cookies	0.69 (0.01)	1.95 (0.03)	0.54 (0.03)	0.74 (0.04)	0.55 (0.03)	0.76 (0.07)	0.53 (0.01)	< 0.001
Fatty and sweet products	20.37 (0.08)	24.93 (0.27)	14.46 (0.21)	26.63 (0.29)	25.04 (0.23)	28.56 (0.54)	18.73 (0.12)	< 0.001
Yoghurts	58.14 (0.21)	45.32 (0.68)	74.09 (0.54)	61.45 (0.74)	62.92 (0.58)	74.27 (1.36)	53.25 (0.30)	< 0.001
Petit suisse	2.44 (0.04)	1.93 (0.12)	4.04 (0.09)	2.05 (0.13)	1.87 (0.10)	4.09 (0.23)	2.18 (0.05)	< 0.001
Dairy desserts	35.16 (0.15)	34.18 (0.46)	34.00 (0.37)	78.92 (0.51)	36.20 (0.40)	36.77 (0.93)	28.50 (0.20)	< 0.001
White cheeses	17.35 (0.12)	12.54 (0.39)	21.07 (0.31)	14.05 (0.43)	15.76 (0.34)	25.71 (0.79)	17.67 (0.17)	< 0.001
Sweet breakfast cereals and cereal bars	5.54 (0.05)	5.33 (0.15)	5.12 (0.12)	5.96 (0.16)	4.26 (0.13)	6.30 (0.29)	5.93 (0.06)	< 0.001
Breakfast cereals with little sugar, wholemeal foods	2.20 (0.03)	1.49 (0.09)	2.36 (0.07)	1.54 (0.10)	1.41 (0.08)	2.54 (0.18)	2.57 (0.04)	< 0.001
Low sugar breakfast cereals	0.77 (0.01)	0.68 (0.04)	0.77 (0.03)	0.60 (0.05)	0.50 (0.04)	0.67 (0.09)	0.89 (0.02)	< 0.001
Delicatessen	19.62 (0.07)	19.37 (0.23)	16.22 (0.19)	19.09 (0.26)	20.96 (0.20)	21.91 (0.47)	20.33 (0.10)	< 0.001
Dressings, sauces	17.38 (0.05)	17.24 (0.16)	18.37 (0.12)	17.65 (0.17)	24.17 (0.14)	18.22 (0.32)	15.28 (0.07)	< 0.001
Miscellaneous	9.72 (0.03)	9.10 (0.10)	10.16 (0.08)	8.80 (0.10)	8.86 (0.08)	12.83 (0.19)	9.93 (0.04)	< 0.001
High-protein meal replacements and nutritional supplements	1.61 (0.06)	1.10 (0.19)	4.26 (0.15)	1.73 (0.21)	0.83 (0.17)	4.04 (0.39)	0.97 (0.08)	< 0.001

Weighted according to the French national census report data by using the CALMAR macro run by sex and based on age, socio-professional category and housing area^68^.

Mean (SD), adjusted for energy intake and number of 24 h dietary records.

**P values for comparisons between clusters using linear regression.

#### Cluster 1: Consumers of additives found in cookies and sweet cakes

This cluster constituted 9.8% of the study sample. Participants from this cluster presented notably the highest intakes of e322 lecithins, e471 mono-and diglycerides of fatty acids, e500 sodium carbonates, e450 diphosphates, e503 ammonium carbonates, e422 glycerol and e420 sorbitol. Participants from this cluster displayed the highest proportion of postgraduate and non-smoker individuals and had the lowest mean BMI. They had the highest caloric and lipid intakes, but the lowest protein, alcohol and sodium intakes. They were the highest consumers of fatty and sweet cakes and cookies (consistent with their higher intakes of lecithins, mono-and diglycerides of fatty acids, sodium carbonates and glycerol) and fatty and salty products.

#### Cluster 2: Consumers of additives found in broths, meal substitutes, butter and bread

This cluster constituted 14.7% of the sample. They were the highest consumers of e14xx modified starches, e621 monosodium glutamate, e304 fatty acid esters of ascorbic acid and e320 butylated hydroxyanisole (BHA). Participants from this cluster were notably the oldest and the most physically active, with the lowest proportion of current smokers. They had the highest intake of sodium, the lowest lipid intake and were among the highest consumers of butter and margarines, meal substitutes and broth (consistent with the high intakes of monosodium glutamate and BHA).

#### Cluster 3: Consumers of additives found in dairy desserts, breakfast cereals and pastries

This cluster constituted 8.4% of the sample. They were notably the highest consumers of e407 carrageenan, e270 lactic acid, e282 calcium propionate, e452 polyphosphates, e160b annatto, e1442 hydroxy propyl distarch phosphate. These participants had relatively high carbohydrate intakes. They were the highest consumers of dairy desserts, which could explain the high intakes of carrageenans, lactic acid and hydroxy propyl distarch phosphate. They were also high consumers of pastries (consistent with higher intakes of calcium propionate), sweetened breakfast cereals and cereal bars.

#### Cluster 4: Consumers of additives found in industrial sauces and processed meat

This cluster constituted 12.3% of the sample. They notably had the highest intakes of e250 sodium nitrite, e316 sodium erythorbate, e451 triphosphates and e120 cochineal which are particularly used in processed meat. They also had the highest intakes of e330 citric acid, e415 xanthan gum, e202 potassium sorbate, e412 guar gum, e224 potassium metabisulphite and e150a plain caramel. This cluster included the highest proportion of men and had an overall lower level of education. They had the lowest carbohydrate intake. They were the highest consumers of bread, fish, rice, semolina, dressings and sauces (the latest being consistent with higher intakes of e415 xanthan gum, e202 potassium sorbate, e412 guar gum, e224 potassium metabisulphite). They were also high consumers of processed meat and pork and poultry hams (consistent with higher intakes of sodium nitrite, sodium erythorbate, triphosphates and cochineal).

#### Cluster 5: Consumers of additives found in sugary and artificially sweetened beverages

This cluster constituted 2.6% of the sample. They were notably the highest consumers of the 4 main sweeteners (e950 acesulfame K, e951 aspartame, e955 sucralose, e960 steviol glycosides), and of e440 pectins, e160a carotenes, e331 sodium citrates, e301 sodium ascorbate, e160c paprika extract, e150d sulphite ammonia caramel, e100 curcumin, e252 potassium nitrate, e338 phosphoric acid, e161b lutein, e211 sodium benzoate, e472 esters of mono- and diglycerides and e212 potassium benzoate. These participants had higher BMI, were the youngest, had the lowest physical activity and were more likely to be smokers. They had an intermediate caloric intake, the highest protein and UPF intakes, and the lowest proportion of organic food in their diet. They were notably the highest consumers of non-alcoholic sweetened and unsweetened drinks (including sugary and artificially sweetened sodas, in line with higher intakes of sweeteners, sodium and potassium benzoates, sodium citrates, phosphoric acid and sulphite ammonia caramel), processed meat, pork and poultry hams (consistent with higher intakes of potassium nitrate), and table-top sweeteners in powder.

#### Cluster 6: Consumers of various staple foods with low additive content

This cluster constituted 52.1% of the study sample, with the highest proportion of women (74.3%). It presented the lowest mean intakes for all food additives. It was characterized by its lower caloric intake, higher proportion of organic food and lower proportion of UPF in the diet, and higher alcohol intake. Participants of this cluster were high consumers of "staple foods": whole-grain products, pulses, breakfast cereals with little or no added sugar, vegetable juice, oleaginous fruits, vegetable oils, and cheese.

## Discussion

To our knowledge, this large population-based study was the first to estimate chronic exposure to food additive mixtures based on detailed consumption and composition data for a wide range of substances. Forty-eight additives were consumed by more than 10% of the participants, with modified starches and citric acid consumed by more than 90%. The top 50 also included several food additives for which potential adverse health effects have been suggested by recent experimental studies. We identified and described five clusters of participants more specifically exposed to five additive mixtures and one additional cluster gathering participants with overall low additive exposure.

Since 2012, EFSA has started the re-evaluation of all food additives authorized before January 2009. The agency's opinions on an additive are subject to change as evidenced by the update on TiO_2_, which is no longer considered as safe. EFSA has carried out simulations of exposure, combining average food consumption data from European member states with doses of additives reported by manufacturers, for additives and countries for which such data were available. Overall, when comparing exposure estimates with EFSA's, intakes of the NutriNet-Santé population were relatively lower. For instance, for modified starches, we estimated a mean intake of 24.33 mg/kg bodyweight per day (95th percentile: 66.4 mg/kg), versus 112.0 mg/kg bodyweight/day (95th percentile: 235.5 mg/kg) in EFSA's non-brand-loyal scenario (French population group)^76^. Similarly for lecithins: 0.83 mg/kg bodyweight per day (95th percentile: 2.6 mg/kg), versus 6.0 mg/kg bodyweight/day (95th percentile: 13.0 mg/kg)^77^. This overall lower exposure may in part be due to the more health-conscious profiles of NutriNet-Santé participants. However, this may also be due to methodological differences: in the present study, presence/absence of food additives was precisely determined based on the commercial brand and the precise list of ingredients, whereas EFSA stimulations use an average information by product category. Some other studies performed intake estimations for several specific food additives, in particular nitrites/nitrates, colors, monosodium glutamate and sulfites^33,78–92^. Although comparisons of different populations are not straightforward, some similarities in the exposure estimates were observed. For instance, in China, similar mean intakes of monosodium glutamate were found: mean (SD): 2.2 (1.6) g/d^33^ versus 2.4 (20.2) in the present study. However, as for EFSA simulations and except in rare cases, these studies were based on generic food data (not accounting for the specific brand consumed and thus the precise ingredient list). Besides, these studies focused on one specific additive or a very limited number of additives, which did not permit the investigation of mixtures. A recent study by the French food observatory “*Observatoire de l’Alimentation*” (Oqali) evaluated the occurrence of certain food additives in a selection of food products of the French market^93^. The additives most frequently found where consistent with the most consumed in our study (e.g. citric acid, modified starches and lecithins in the top 3; acesulfame K as the most used/consumed sweetener).

For several food additives widely consumed in this study, potential adverse health effects have been suggested by recent *in-vivo*/*in-vitro*, and-rarely-epidemiological studies. For instance, an experiment in humans demonstrated that phosphatidylcholine found in lecithin is converted by bacteria in the gut into trimethylamine-N-oxide, which may potentially contribute to hardening of the arteries or atherosclerosis and heart attack^94^. A potential role in the development of Chron's disease has also been suggested for lecithins^95–97^, and an experimental study among humans suggests a link between lecithins and coronary artery disease through the production of a proatherosclerotic metabolite, trimethylamine-N-oxide (TMAO)^94^. In a study on *ex-vivo* models of human microbiota, 20 emulsifiers were tested and a large majority (including carboxymethylcellulose, polysorbate 80, carrageenans, guar/xanthan gums, lecithins), were able to directly modify the gut microbiota in a way that could promote gut inflammation^23^. Carrageenans have been linked to fasting hyperglycemia and exacerbated glucose intolerance and hyperlipidemia without effect on weight in mice^31^. Also, sodium nitrite and potassium nitrate intakes have been associated in prospective cohorts with all-cause mortality (nitrates/nitrites from preserved/processed meat)^24^, and colorectal, gastric and pancreatic cancers^25–27,98^, although their impact remains debated. Phosphates have been associated with vascular effects (e.g. endothelial dysfunction and vascular calcification) in experimental studies among humans^41,42^. Sulfites have been associated with alteration of the gut and mouth microbiome *in-vitro* at concentrations close to those found in foods^99^. The effects of non-nutritive sweeteners such as acesulfame K, sucralose and aspartame on human cardiometabolic health and adiposity are controversial^37^, and these additives have been linked with hematopoietic neoplasia and gut microbiota alteration in experimental studies on rodents^21,38–40^. Sulfite ammonia caramel, present in almost every cola sodas, might carry 4-methylimidazole (4-MEI) defined as possibly carcinogenic to humans by the International Agency for Research on Cancer (IARC). Monosodium glutamate might have patho-physiological and toxicological effects on human health^32,34^ and was associated with overweight in a prospective cohort^33^. Carboxymethylcellulose has been associated with changes in microbiota composition, intestinal inflammation and metabolic syndrome (*in-vivo*)^43,100–102^, pro-inflammation (*in-vivo*, *ex-vivo*)^46,103–106^ and promotion of tumor development (*in-vivo*)^45^. In mouse models, it was recently shown that in the presence of intestinal inflammation, the food additive ethylenediaminetetraacetate (EDTA) was capable of exacerbating inflammation and inducing colorectal carcinogenesis at doses presumed to be safe^107^.

The NMF procedure followed by k-means clustering allowed us to describe profiles of exposure to mixtures of food additives, which corresponded to specific socio-demographic profiles and dietary behaviors. Although about half of the population study pertained to cluster 6 and tended to have a relatively limited exposure to food additives overall. The other half of the study population was exposed to different additive mixtures (5 main mixtures identified). In a previous work consisting in the exploration of the Open Food Facts database, we identified clusters of additives found in food products of the French market^30^. The mixtures of food additives identified in the present work resulted 1) from the co-occurrence of several additives in a same industrial product (as shown previously^30^) and 2) from the co-consumption of various food products within usual dietary patterns. For instance, participants of cluster 1 were notably the highest consumers of sweet cakes and cookies, thus, they were particularly exposed to food additives of a specific cluster in our previous work ("stabilizers and emulsifiers mostly used in biscuits and cakes").

So far, detailed information on potential cocktail effects of food additives is lacking. However, several studies started to suggest potential interactions and synergies. For instance, mixture of colorings with sodium benzoate were associated with increased hyperactivity in children^108^. Neurotoxic effects were also observed between combinations of brilliant blue with L-glutamic acid and quinoline yellow with aspartame *in-vitro*^109^ and a mixture of food coloring additives increased oxidative stress in rats^110^. Future prospective studies and experimental research should investigate the health effects of chronic exposure to these mixtures of food additives, as they are consumed in real life.

Strengths of this study included the large sample size and the accuracy of dietary intake data used to estimate additive exposure at the individual level, which is necessary for future etiological studies (population-based simulations are not appropriate for this purpose). Indeed, repeated 24 h records allowed us to collect detailed information on > 3500 generic foods/beverages, each declined in dozens of commercial brands, which is a strength compared to previous nutritional studies. Three complementary databases were used to determine qualitative additive composition and thousands of assays were performed and complemented by EFSA and GSFA data to retrieve information on quantitative doses. However, some limitations should be acknowledged. First, not all food additives could be covered due to a lack of quantitative data for some additives. However, the latter were not the most relevant in terms of potential public health impact since they were mostly consumed by less than 10% of the population. Second, as it is generally the case for cohorts with a primary etiological focus, the recruitment method was based on a voluntary participation and the study population was not intended to be representative of the French population. Thus, the individuals included in the cohort were more often women, with "healthier" behaviors, a higher socioeconomic status and a higher level of education than the general French population^111,112^. However, even if lowest socioeconomic statuses were under-represented, the cohort still included about 6% of unemployed citizens or state aid recipients, which is lower than the national ≈10%, but higher than in other health studies that are not Internet-based. Moreover, the geographical distribution of the cohort was close to that of metropolitan France^113^. Also, the proportion of energy intake brought by ultra-processed foods (i.e. the main sources of food additives) among the participants of the cohort was 30–35%, consistent with the 31% assessed in two French nationally representative surveys^114,115^. Besides, a potential selection bias has been minimized since all analyses were weighted according to the characteristics of the French population (INSEE 2016 census). Last, industrial products may be reformulated across time by choice of manufacturers or regulation requirements, thereby complicating exposure assessment. However, bias linked to this aspect was limited in the present study since 1) the composition and consumption data were matched taking into account the year (dynamic matching), accounting for different compositions for a same product/brand consumed several years apart; and 2) the top 50 of most consumed food additives computed for 3 different periods of time in the 2009–2020 time-frame marginally changed, which illustrates the relative stability of additive exposure. In future etiological studies, it will be possible to study food additive exposure as time-dependent variables.

This large population-based study provided for the first time a comprehensive overview of intakes for a wide range of additives, highlighting a widespread consumption of food additives for which health concerns are currently debated, and identified mixtures of food additives that were associated to consumer and food consumption profiles. Their health impact and potential cocktail effects should be explored in future epidemiological and experimental studies. In the meantime, and following the precautionary principle, several public health authorities worldwide recently started to recommend limiting the consumption of ultra-processed foods and, in practice, choosing food products of better nutritional quality (according to the Nutri-Score^116^) and without or with as few additives as possible^117,118^.

## Supplementary Information

Supplementary Information.

## Data Availability

Data described in the manuscript, code book, and analytic code will be made available upon request pending application and approval. Researchers from public institutions can submit a collaboration request including information on the institution and a brief description of the project to collaboration@etude-nutrinet-sante.fr. All requests will be reviewed by the steering committee of the NutriNet-Santé study. A financial contribution may be requested. If the collaboration is accepted, a data access agreement will be necessary and appropriate authorizations from the competent administrative authorities may be needed. In accordance with existing regulations, no personal data will be accessible.

## Abbreviations

ADI: Acceptable Daily Intake
EFSA: European Food Safety Authority
GNPD: Global New Products Database
IPAQ: International Physical Activity Questionnaire
GSFA: General Standard for Food Additives
NMF: Non-negative Matrix Factorization
OQALI: Observatoire de la Qualité de l'Alimentation
