# Supplementary material for: Exposure to food additive mixtures in 106,000 French adults from the NutriNet-Santé cohort
Source: Sci Rep. 2021 Oct 4;11:19680. doi: 10.1038/s41598-021-98496-6 (PMC8490357; doi:10.1038/s41598-021-98496-6)
Supplement: Supplementary file 1 — Supplementary Information. [file 41598_2021_98496_MOESM1_ESM.docx]

Exposure to food additive mixtures in 106,000 French adults from the NutriNet-Santé cohort

## APPENDIX 1: Computation of food additive data

## Description of the databases for qualitative composition information

### Open Food Facts

Open Food Facts (OFF) (<http://world.openfoodfacts.org/>) is an open collaborative database of food products marketed worldwide under the Open Database License (ODBL). This French initiative contains data on hundreds of thousands of products. The initiative started in France in 2012, offering extensive coverage of the French food market, and a growing number of products are available for other countries around the world. Contributors (citizens and active OFF contributors) are constantly adding products to this popular database by scanning the barcode and sending photographs of the packaging. The information is processed automatically by artificial intelligence to retrieve different information for each product, such as trade name, brand, list of ingredients (including food additives) and nutritional composition. As food product formulations may change, compositions are regularly updated when they are re-registered by consumers. Each product is assigned one or more food categories and is identified by the Global Trade Item Number (GTIN) embedded in the barcode.

### OQALI

Oqali (French Food Quality Observatory, <https://www.oqali.fr/oqali_eng/>) has been carrying out studies to characterize the nutritional quality of the food supply of processed products, both in terms of the information available on the packaging and the nutritional composition of the products. It is jointly implemented by Anses (Agence nationale de sécurité sanitaire de l’alimentation, de l’environnement et du travail) and INRAE (Institut National de la Recherche pour l'Agriculture, l'alimentation et l'Environnement). The database includes 49,854 products (studied between 2008 and 2019) from 30 food sectors and from 5 market segments (specialist retailers, national brands, private labels, entry-level private labels and hard discount), identified by barcode. The database contains information on ingredient list, including food additives.

### GNPD

Mintel Global New Products Database (GNPD) is an online international database that registers food innovations (new product launched on the market, reformulation or packaging changes). Over five million records from more than 80 countries provide product ingredients, nutrition facts, packaging, distribution and pricing information. After extraction of the database in 2019, detection of food additives labels or numbers was performed based on the ingredient list.

## Merging NutriNet-Santé food consumption data with and Open Food Facts, Oqali and GNPD composition data

First, NutriNet-Santé generic food or beverage items were merged with the OFF categories by a trained team of three dietitians, one data-manager and one computer scientist. Only the OFF products with an available ingredient list were kept. After elimination of staple items generally consumed without brand names (such as fresh vegetables), 1557 generic NutriNet food items (e.g. “chocolate cookie”) were matched with OFF categories. Two types of matches were performed:

- Direct matches between a generic NutriNet item and an OFF category (for 676 NutriNet items)

- More complex matches (with multiple categories or a category and a keyword). Example: the NutriNet item "Plain Cereal Bars" was matched with the OFF products of the category "Cereal Bars" and containing the keyword "plain" in their name.

Then, NutriNet-Santé and OFF food items were matched at the brand/commercial name level. Indeed, in NutriNet-Santé 24h dietary records, participant declare the brand of the product consumed by checking from a list of predefined brands or by typing in plain text. A table of correspondence between the predefined brands in NutriNet and the different OFF brands (n=20,000) was therefore created by the dietitians. For the brands entered in clear text, each entry was cleaned and matched with an OFF brand. When the brand was missing, imputations were made considering (in the following order):

1) the brand most frequently declared by the participant for the same NutriNet food item (in other meals of other days of 24-hour records)

2) participant's answers to a questionnaire specifically designed to collect information on most frequently consumed brands for each product category

3) the brand most declared by the Nutrinaute (as in 1), but based on the food category of the brand questionnaire (which contains several NutriNet items).

Similarly, NutriNet-Santé data was matched with the OQALI and GNPD databases.

When several industrial food products of food additive composition are possible candidates for a commercial food declared in NutriNet, databases were prioritized as follows: Oqali (national and official database from the French food safety agency), then Open Food Facts (very wide coverage of the French market) and lastly GNPD (as an international database, ingredients are registered in English which may lower the sensitivity of additive detection in the list). Moreover, to account for possible reformulations, products with a date of inclusion in the databases within +/- 1 year of the date of consumption were considered (dynamic matching).

## Quantitative data

No comprehensive quantitative database was available because information on the amount of each additive in a given product is not mentioned on its label. The quantitative composition of additives has therefore been derived from several sources. Firstly, ad-hoc laboratory assays have been carried out, prioritizing the most consumed additives and those with suspected health effects. The selected food products were the main vectors of these additives in our study population. 2677 assays were performed. These assays were performed by Mérieux and Eurofins firms and the French DGCCRF public laboratories. We also retrieved data from ad hoc dosing previously commended by the association "UFC Que Choisir" which covered 39 additives and 1721 products. These data from laboratory assays were used to impute doses: e.g. for a NutriNet food item of a specific brand that contains a specific additive but for which no assay data was available, all values of assays corresponding to the same generic item but to other brands were averaged. Besides, during the re-evaluation of food additives, the European Food Safety Authority (EFSA) carries out exposure simulations based on the doses of additives in food products communicated by manufacturers across Europe. These doses (at the generic food item level – no brand data) have been used when no dose was available from laboratory assays. When EFSA data was missing on usage doses, the maximum levels authorized by the regulation were used^1^. Last, quantitative additive data from the Codex General Standard for Food Additives (GSFA)^2^ were used. Figure 1 illustrates the decision tree for the computation of food additive doses.


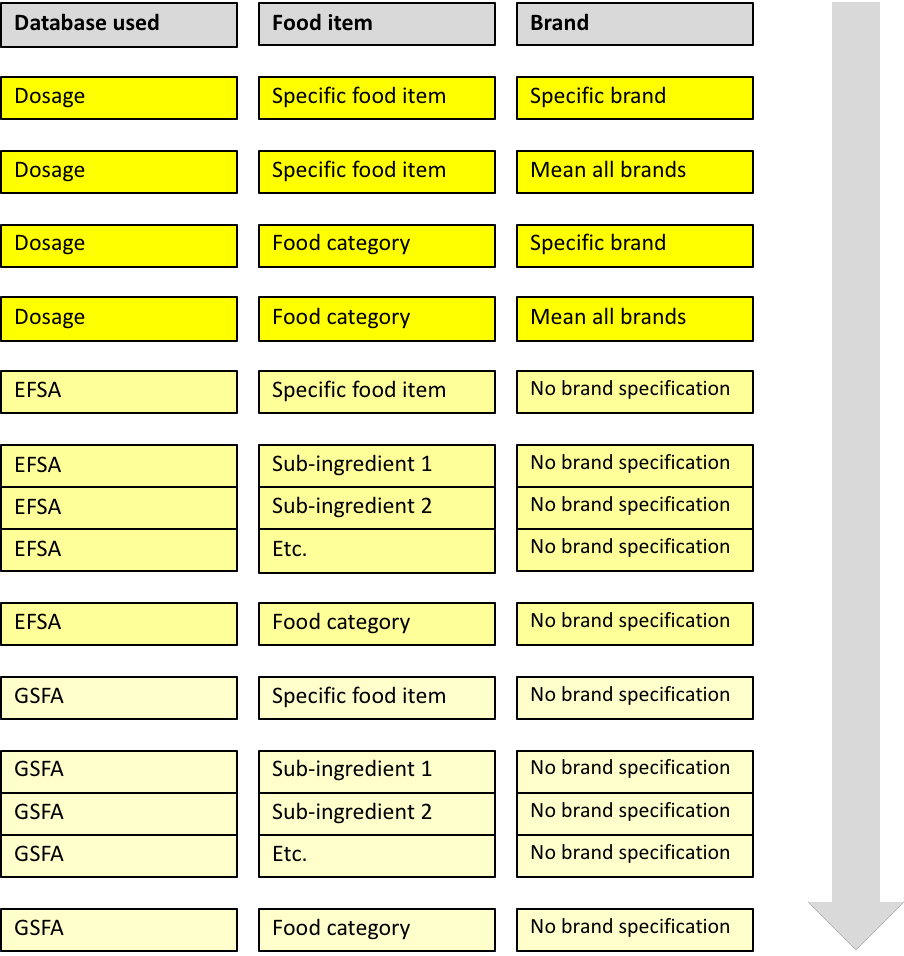


Figure 1: Decision tree for food additive doses computation

## APPENDIX 2: Nonnegative matrix factorization methodology

The aim of Nonnegative Matrix Factorization (NMF) is the factorization of a matrix, allowing to approximate its original information as precisely as possible, but with a reduced number of components^3,4^. Compared to other factorization methods such as principal component analysis (PCA), its particularity is the constraint of nonnegative values (both for the original data and for the outputted components), and that no independence constraints are imposed to the components. It is particularly adapted for sparse matrices such as ours, where individuals are described by a lot of food additives variables with many zeroes, and its prerequisite of nonnegative data is not problematic in the case of food consumption data. Also, compared with other factorization methods, the absence of independence of components is interesting for food data approximation as an individual can combine several components generated by the NMF, which better takes into account the diversity of consumption behaviors within a population^5^.

Given a nonnegative matrix *A*, NMF seeks to produce rank-*k* matrices *W* and *H* such as:

$$A \approx W H$$

With *W* and *H* nonnegative and:

$$k \ll rank(A)$$

Each column of *X* is thus approximated by a non-negative linear combination of the columns of *W* (the basis components), where the coefficients are given by the corresponding column of *H* (the mixture coefficients).

The NMF algorithms estimates *W* and *H* as a local minimum of the following optimization problem:

$${min}_{W, H\geq0}\left[ D(X, WH) + R(W, H) \right]$$

With *D* a loss function that measures the approximation quality, and *R* an optional penalization function designed to improve desirable properties on matrices *W* and *H*. *D* can be a least squares criterion (Frobenius norm) or the Kullback-Leibler divergence.

The algorithms used and the number of ranks *k* can be determined after evaluation of several measures, such as the quality of estimation (e.g. residuals, part of explained variance and sparseness^6^) or the stability after multiple runs (e.g. cophenetic correlation coefficient^7^). It is then possible to use the generated matrices *W* and *H* in classifications such as k-means.

## APPENDIX 3: Flowchart for the selection of the study population, NutriNet-Santé cohort, France 2009-2020 (N=106,489)

##
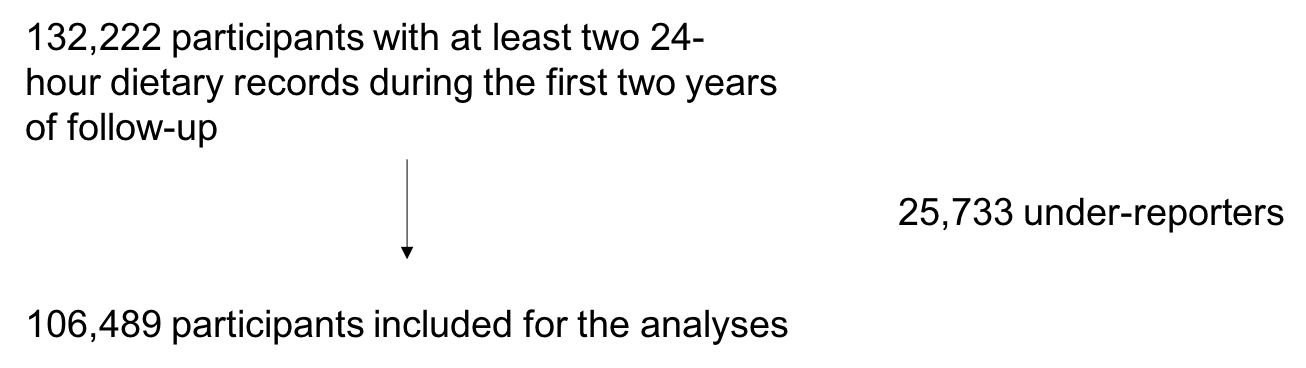


## APPENDIX 4: Top 50 of the most frequently consumed food additives according to percent of consumers, NutriNet-Santé cohort, France 2009-2020 (N=106,489), across three different consumption periods

## Period 1: 2009 - 2013

| Food additive | Percent of consumers | Median (mg/day) | 95th percentile (mg/day) | Mean (SD) (mg/day) | Median (mg/kg bw/day) | 95th percentile (mg/kg bw/day) | Mean (SD) (mg/kg bw/day) | ADI  (mg/kg bw/day) | % of participants exceeding the ADI | % of participants exceeding the ADI (among consumers) |
| --- | --- | --- | --- | --- | --- | --- | --- | --- | --- | --- |
| E 14xx Modified Starches | 92.00 | 1313.20 | 4238.90 | 1596.67 (1379.17) | 19.90 | 65.73 | 24.24 (21.4) | NA | NA | NA |
| E 330 Citric acid | 91.71 | 1413.85 | 6220.03 | 1980.8 (2227.15) | 21.21 | 95.73 | 30.17 (34.19) | NA | NA | NA |
| E 322 Lecithins | 86.91 | 36.00 | 168.89 | 54.49 (72.29) | 0.54 | 2.64 | 0.84 (1.11) | NA | NA | NA |
| E 300 Ascorbic acid | 80.96 | 6.20 | 34.86 | 10.45 (16.11) | 0.09 | 0.54 | 0.16 (0.26) | NA | NA | NA |
| E 415 Xanthan gum | 80.23 | 314.75 | 1419.00 | 447.22 (513.62) | 4.73 | 21.37 | 6.72 (7.67) | NA | NA | NA |
| E 440 Pectins | 79.71 | 116.11 | 714.29 | 205.2 (307.47) | 1.74 | 10.90 | 3.11 (4.7) | NA | NA | NA |
| E 471 Mono-and diglycerides of fatty acids | 79.11 | 105.00 | 556.67 | 164.28 (199.06) | 1.59 | 8.50 | 2.49 (3) | NA | NA | NA |
| E 407 Carrageenan | 78.39 | 19.98 | 178.51 | 47.03 (69.89) | 0.30 | 2.69 | 0.71 (1.06) | 75 | 0 | 0 |
| E 250 Sodium nitrite | 75.76 | 0.12 | 0.87 | 0.23 (0.34) | 0.00 | 0.01 | 0 (0) | 0.07 | 0.01 | 0.02 |
| E 412 Guar gum | 72.27 | 200.79 | 1102.22 | 321.32 (405.92) | 3.02 | 16.54 | 4.81 (6.02) | NA | NA | NA |
| E 500 Sodium carbonates | 67.23 | 555.00 | 4850.00 | 1252.92 (1871.23) | 8.47 | 75.22 | 19.35 (28.99) | NA | NA | NA |
| E 202 Potassium sorbate | 66.69 | 6.69 | 82.70 | 19.98 (34.51) | 0.11 | 1.25 | 0.3 (0.51) | 3 | 0.16 | 0.23 |
| E 450 Diphosphates | 64.91 | 63.11 | 707.63 | 176.36 (304.16) | 0.95 | 10.94 | 2.71 (4.6) | 40 | 0.01 | 0.02 |
| E 316 Sodium erythorbate | 51.41 | 0.36 | 32.74 | 6.83 (14.81) | 0.01 | 0.46 | 0.1 (0.22) | 6 | 0 | 0 |
| E 160c Paprika extract, capsanthin, capsorubin | 50.72 | 0.00 | 0.89 | 0.18 (0.39) | 0.00 | 0.01 | 0 (0.01) | 24 | 0 | 0 |
| E 301 Sodium ascorbate | 50.15 | 0.00 | 29.14 | 6.66 (12.26) | 0.00 | 0.43 | 0.1 (0.18) | NA | NA | NA |
| E 331 Sodium citrates | 46.53 | 0.00 | 412.50 | 87.32 (222.92) | 0.00 | 6.10 | 1.3 (3.27) | NA | NA | NA |
| E 224 Potassium metabisulphite | 46.22 | 0.00 | 8.37 | 1.53 (5.11) | 0.00 | 0.13 | 0.02 (0.07) | 0.7 | 0.11 | 0.23 |
| E 160a Carotenes | 44.71 | 0.00 | 14.66 | 2.26 (8.87) | 0.00 | 0.22 | 0.03 (0.13) | NA | NA | NA |
| E 503 Ammonium carbonates | 44.23 | 0.00 | 2400.00 | 494.42 (1103.68) | 0.00 | 36.89 | 7.53 (16.29) | NA | NA | NA |
| E 160 Carotene | 42.58 | 0.00 | 1.26 | 0.4 (3.44) | 0.00 | 0.02 | 0.01 (0.05) | NA | NA | NA |
| E 410 Locust bean gum | 39.95 | 0.00 | 550.00 | 119.58 (234.05) | 0.00 | 8.21 | 1.81 (3.55) | NA | NA | NA |
| E 950 Acesulfame K | 35.91 | 0.00 | 24.12 | 4.65 (15.21) | 0.00 | 0.35 | 0.07 (0.23) | NA | NA | NA |
| E 120 Cochineal, Carminic acid, Carmines | 34.80 | 0.00 | 1.96 | 0.37 (1.47) | 0.00 | 0.03 | 0.01 (0.02) | 5 | 0 | 0 |
| E 100 Curcumin | 34.37 | 0.00 | 6.43 | 0.99 (3.55) | 0.00 | 0.09 | 0.01 (0.05) | NA | NA | NA |
| E 270 Lactic acid | 34.35 | 0.00 | 10.47 | 1.86 (4.92) | 0.00 | 0.16 | 0.03 (0.07) | NA | NA | NA |
| E 252 Potassium nitrate | 32.33 | 0.00 | 0.94 | 0.18 (0.43) | 0.00 | 0.01 | 0 (0.01) | 3.7 | 0 | 0 |
| E 150d Sulphite ammonia caramel | 29.86 | 0.00 | 620.54 | 105.34 (346.75) | 0.00 | 9.14 | 1.56 (5.15) | 300 | 0 | 0 |
| E 951 Aspartame | 29.61 | 0.00 | 53.34 | 9.29 (31.79) | 0.00 | 0.77 | 0.14 (0.49) | 40 | 0 | 0 |
| E 420 Sorbitols | 28.58 | 0.00 | 282.45 | 48.85 (155.8) | 0.00 | 4.07 | 0.75 (2.36) | NA | NA | NA |
| E 161b Lutein | 26.32 | 0.00 | 0.79 | 0.42 (3.21) | 0.00 | 0.01 | 0.01 (0.05) | 1 | 0.04 | 0.14 |
| E 422 Glycerol | 25.41 | 0.00 | 796.42 | 139.91 (485.24) | 0.00 | 12.39 | 2.18 (7.43) | NA | NA | NA |
| E 392 Extracts of rosemary | 24.41 | 0.00 | 6.30 | 0.97 (2.99) | 0.00 | 0.09 | 0.01 (0.04) | 0.3 | 0.32 | 1.33 |
| E 282 Calcium propionate | 23.11 | 0.00 | 57.14 | 9.75 (28.85) | 0.00 | 0.91 | 0.15 (0.44) | NA | NA | NA |
| E 150a Plain caramel | 23.10 | 0.00 | 47.98 | 7.35 (31.2) | 0.00 | 0.73 | 0.11 (0.47) | 300 | 0 | 0 |
| E 451 Triphosphates | 22.20 | 0.00 | 267.86 | 45.37 (141.85) | 0.00 | 4.11 | 0.67 (2.09) | 40 | 0.01 | 0.01 |
| E 452 Polyphosphates | 21.55 | 0.00 | 180.57 | 36.48 (170.67) | 0.00 | 2.69 | 0.54 (2.57) | 40 | 0.01 | 0.02 |
| E 338 Phosphoric acid | 21.11 | 0.00 | 81.66 | 13.37 (45.84) | 0.00 | 1.20 | 0.2 (0.68) | NA | NA | NA |
| E 160b Annatto, Bixin, Norbixin | 20.04 | 0.00 | 0.52 | 0.08 (0.28) | 0.00 | 0.01 | 0 (0) | 0.3 | 0 | 0 |
| E 220 Sulphur dioxide | 17.21 | 0.00 | 0.37 | 0.15 (1.39) | 0.00 | 0.01 | 0 (0.02) | 0.7 | 0.01 | 0.06 |
| E 1442 Hydroxy propyl distarch phosphate | 16.45 | 0.00 | 416.67 | 58.49 (200.3) | 0.00 | 5.95 | 0.89 (3.16) | NA | NA | NA |
| E 472e Mono- and diacetyl tartaric acid esters of mono- and diglycerides of fatty acids | 15.88 | 0.00 | 96.14 | 15.3 (60.81) | 0.00 | 1.47 | 0.23 (0.93) | NA | NA | NA |
| E 621 Monosodium glutamate | 15.47 | 0.00 | 4513.38 | 2316.33 (19985.64) | 0.00 | 71.54 | 35.68 (306.2) | 30 | 7.14 | 46.02 |
| E 162 Beetroot Red, betanin | 14.94 | 0.00 | 4.57 | 0.75 (3.09) | 0.00 | 0.07 | 0.01 (0.05) | NA | NA | NA |
| E 476 Polyglycerol polyricinoleate | 14.36 | 0.00 | 22.12 | 3.56 (16.25) | 0.00 | 0.32 | 0.05 (0.25) | 25 | 0 | 0 |
| E 341 Calcium phosphates | 13.94 | 0.00 | 87.30 | 23.46 (207.84) | 0.00 | 1.36 | 0.37 (3.47) | 40 | 0.1 | 0.72 |
| E 955 Sucralose | 13.90 | 0.00 | 6.86 | 1.66 (15.34) | 0.00 | 0.10 | 0.02 (0.25) | NA | NA | NA |
| E 163 Anthocyanins | 12.80 | 0.00 | 7.29 | 1.27 (7.01) | 0.00 | 0.11 | 0.02 (0.11) | NA | NA | NA |
| E 466 Sodium carboxy methyl cellulose, Cellulose gum | 12.60 | 0.00 | 123.81 | 20.65 (96.98) | 0.00 | 1.90 | 0.32 (1.52) | NA | NA | NA |
| E 296 Malic acid | 12.18 | 0.00 | 26.79 | 6.85 (52.15) | 0.00 | 0.42 | 0.11 (0.8) | NA | NA | NA |
| Weighted | | | | | | | |  |  |  |

## Period 2: 2013 - 2017

| Food additive | Percent of consumers | Median (mg/day) | 95th percentile (mg/day) | Mean (SD) (mg/day) | Median (mg/kg bw/day) | 95th percentile (mg/kg bw/day) | Mean (SD) (mg/kg bw/day) | ADI  (mg/kg bw/day) | % of participants exceeding the ADI | % of participants exceeding the ADI (among consumers) |
| --- | --- | --- | --- | --- | --- | --- | --- | --- | --- | --- |
| E 14xx Modified Starches | 89.28 | 1337.85 | 4640.73 | 1676.68 (1508.91) | 20.89 | 73.92 | 26.24 (24.13) | NA | NA | NA |
| E 330 Citric acid | 87.86 | 1138.58 | 6020.36 | 1799.51 (2274) | 17.57 | 91.88 | 27.9 (35.87) | NA | NA | NA |
| E 322 Lecithins | 84.66 | 30.84 | 166.69 | 50.2 (65.42) | 0.48 | 2.63 | 0.78 (1.03) | NA | NA | NA |
| E 300 Ascorbic acid | 78.53 | 6.06 | 36.35 | 10.59 (16.76) | 0.10 | 0.58 | 0.17 (0.26) | NA | NA | NA |
| E 415 Xanthan gum | 77.76 | 251.62 | 1240.80 | 379.27 (457.26) | 3.88 | 18.87 | 5.86 (7.07) | NA | NA | NA |
| E 440 Pectins | 75.26 | 87.90 | 580.80 | 162.65 (255.86) | 1.39 | 8.84 | 2.53 (4.01) | NA | NA | NA |
| E 407 Carrageenan | 72.83 | 13.75 | 180.18 | 43.09 (69.72) | 0.21 | 2.73 | 0.67 (1.11) | 75 | 0 | 0 |
| E 471 Mono-and diglycerides of fatty acids | 71.80 | 71.43 | 483.33 | 134.02 (177.1) | 1.11 | 7.64 | 2.08 (2.79) | NA | NA | NA |
| E 500 Sodium carbonates | 67.64 | 625.00 | 5732.29 | 1431.7 (2232.53) | 10.00 | 88.68 | 22.57 (35.96) | NA | NA | NA |
| E 412 Guar gum | 62.12 | 102.34 | 809.96 | 217.27 (314.15) | 1.58 | 12.40 | 3.35 (4.85) | NA | NA | NA |
| E 450 Diphosphates | 61.73 | 65.48 | 825.01 | 197.3 (334.94) | 1.00 | 12.72 | 3.08 (5.26) | 40 | 0.07 | 0.11 |
| E 250 Sodium nitrite | 61.25 | 0.07 | 0.75 | 0.19 (0.35) | 0.00 | 0.01 | 0 (0.01) | 0.07 | 0.05 | 0.09 |
| E 202 Potassium sorbate | 61.20 | 3.37 | 70.50 | 16.25 (31.11) | 0.05 | 1.11 | 0.25 (0.48) | 3 | 0.16 | 0.26 |
| E 301 Sodium ascorbate | 44.74 | 0.00 | 28.05 | 5.98 (11) | 0.00 | 0.41 | 0.09 (0.17) | NA | NA | NA |
| E 503 Ammonium carbonates | 40.80 | 0.00 | 2253.33 | 441.03 (1035) | 0.00 | 35.24 | 6.99 (16.45) | NA | NA | NA |
| E 316 Sodium erythorbate | 40.42 | 0.00 | 29.00 | 5.51 (13.42) | 0.00 | 0.43 | 0.09 (0.21) | 6 | 0 | 0 |
| E 160c Paprika extract, capsanthin, capsorubin | 38.51 | 0.00 | 0.64 | 0.12 (0.31) | 0.00 | 0.01 | 0 (0) | 24 | 0 | 0 |
| E 224 Potassium metabisulphite | 36.87 | 0.00 | 6.47 | 1.15 (4.21) | 0.00 | 0.10 | 0.02 (0.06) | 0.7 | 0.12 | 0.31 |
| E 160a Carotenes | 35.79 | 0.00 | 7.33 | 1.37 (7.67) | 0.00 | 0.11 | 0.02 (0.13) | NA | NA | NA |
| E 331 Sodium citrates | 35.75 | 0.00 | 294.64 | 56.5 (164.18) | 0.00 | 4.33 | 0.86 (2.57) | NA | NA | NA |
| E 160 Carotene | 31.77 | 0.00 | 0.87 | 0.37 (3.27) | 0.00 | 0.01 | 0.01 (0.05) | NA | NA | NA |
| E 270 Lactic acid | 31.38 | 0.00 | 11.38 | 1.98 (5.95) | 0.00 | 0.18 | 0.03 (0.09) | NA | NA | NA |
| E 410 Locust bean gum | 27.82 | 0.00 | 368.30 | 65.36 (167.68) | 0.00 | 5.85 | 1.01 (2.65) | NA | NA | NA |
| E 120 Cochineal, Carminic acid, Carmines | 27.56 | 0.00 | 2.01 | 0.44 (2.29) | 0.00 | 0.03 | 0.01 (0.03) | 5 | 0 | 0 |
| E 252 Potassium nitrate | 27.14 | 0.00 | 0.91 | 0.17 (0.46) | 0.00 | 0.01 | 0 (0.01) | 3.7 | 0 | 0 |
| E 422 Glycerol | 26.74 | 0.00 | 952.31 | 174.29 (606.85) | 0.00 | 15.01 | 2.76 (9.59) | NA | NA | NA |
| E 100 Curcumin | 25.53 | 0.00 | 3.21 | 0.55 (2.61) | 0.00 | 0.05 | 0.01 (0.04) | NA | NA | NA |
| E 420 Sorbitols | 24.24 | 0.00 | 257.55 | 43.98 (155.05) | 0.00 | 3.99 | 0.68 (2.36) | NA | NA | NA |
| E 282 Calcium propionate | 24.13 | 0.00 | 66.44 | 11.1 (31.56) | 0.00 | 1.03 | 0.17 (0.5) | NA | NA | NA |
| E 451 Triphosphates | 22.87 | 0.00 | 330.36 | 59.15 (204.22) | 0.00 | 5.08 | 0.91 (3.13) | 40 | 0.08 | 0.13 |
| E 150d Sulphite ammonia caramel | 22.07 | 0.00 | 372.32 | 61.59 (244.08) | 0.00 | 5.43 | 0.93 (3.7) | 300 | 0 | 0 |
| E 150a Plain caramel | 21.60 | 0.00 | 50.02 | 9.05 (42.46) | 0.00 | 0.79 | 0.14 (0.66) | 300 | 0 | 0 |
| E 950 Acesulfame K | 20.79 | 0.00 | 13.76 | 2.43 (10.6) | 0.00 | 0.20 | 0.04 (0.17) | NA | NA | NA |
| E 161b Lutein | 18.79 | 0.00 | 0.32 | 0.4 (3.76) | 0.00 | 0.00 | 0.01 (0.06) | 1 | 0.08 | 0.45 |
| E 452 Polyphosphates | 18.72 | 0.00 | 171.43 | 34.44 (175.01) | 0.00 | 2.60 | 0.53 (2.65) | 40 | 0.07 | 0.11 |
| E 392 Extracts of rosemary | 17.24 | 0.00 | 4.73 | 0.66 (2.43) | 0.00 | 0.07 | 0.01 (0.04) | 0.3 | 0.26 | 1.53 |
| E 160b Annatto, Bixin, Norbixin | 15.65 | 0.00 | 0.36 | 0.06 (0.24) | 0.00 | 0.01 | 0 (0) | 0.3 | 0 | 0 |
| E 341 Calcium phosphates | 15.62 | 0.00 | 111.55 | 24.38 (182.32) | 0.00 | 1.71 | 0.39 (2.85) | 40 | 0.11 | 0.68 |
| E 951 Aspartame | 14.52 | 0.00 | 18.67 | 3.44 (17.19) | 0.00 | 0.28 | 0.05 (0.29) | 40 | 0 | 0 |
| E 262 Sodium acetates | 13.46 | 0.00 | 23.60 | 3.38 (13.37) | 0.00 | 0.35 | 0.05 (0.21) | NA | NA | NA |
| E 338 Phosphoric acid | 13.10 | 0.00 | 45.29 | 7.51 (32.15) | 0.00 | 0.70 | 0.11 (0.49) | NA | NA | NA |
| E 621 Monosodium glutamate | 12.91 | 0.00 | 6095.05 | 2976.91 (22137.99) | 0.00 | 95.43 | 46.61 (357.96) | 30 | 7.06 | 54.75 |
| E 220 Sulphur dioxide | 12.68 | 0.00 | 0.20 | 0.08 (0.72) | 0.00 | 0.00 | 0 (0.01) | 0.7 | 0 | 0 |
| E 472e Mono- and diacetyl tartaric acid esters of mono- and diglycerides of fatty acids | 12.45 | 0.00 | 81.25 | 13.9 (68.56) | 0.00 | 1.29 | 0.22 (1.15) | NA | NA | NA |
| E 296 Malic acid | 12.36 | 0.00 | 41.79 | 7.94 (44.6) | 0.00 | 0.62 | 0.12 (0.69) | NA | NA | NA |
| E 162 Beetroot Red, betanin | 11.44 | 0.00 | 2.80 | 0.49 (2.62) | 0.00 | 0.04 | 0.01 (0.04) | NA | NA | NA |
| E 955 Sucralose | 10.64 | 0.00 | 3.44 | 1.16 (13.18) | 0.00 | 0.05 | 0.02 (0.21) | NA | NA | NA |
| E 414 Gum arabic (acacia gum) | 10.41 | 0.00 | 95.24 | 24.32 (199.84) | 0.00 | 1.59 | 0.38 (3.2) | NA | NA | NA |
| E 1442 Hydroxy propyl distarch phosphate | 10.04 | 0.00 | 223.21 | 32.28 (131.27) | 0.00 | 3.72 | 0.5 (2.11) | NA | NA | NA |
| E 476 Polyglycerol polyricinoleate | 10.03 | 0.00 | 16.29 | 2.83 (15.44) | 0.00 | 0.25 | 0.04 (0.25) | 25 | 0 | 0 |
| Weighted | | | | | | | |  |  |  |

## Period 3: 2017 - 2020

| Food additive | Percent of consumers | Median (mg/day) | 95th percentile (mg/day) | Mean (SD) (mg/day) | Median (mg/kg bw/day) | 95th percentile (mg/kg bw/day) | Mean (SD) (mg/kg bw/day) | ADI  (mg/kg bw/day) | % of participants exceeding the ADI | % of participants exceeding the ADI (among consumers) |
| --- | --- | --- | --- | --- | --- | --- | --- | --- | --- | --- |
| E 330 Citric acid | 82.92 | 885.23 | 5685.60 | 1576.86 (2184.05) | 14.08 | 88.29 | 24.3 (32.44) | NA | NA | NA |
| E 322 Lecithins | 80.99 | 25.39 | 149.47 | 45.37 (82.43) | 0.41 | 2.33 | 0.71 (1.17) | NA | NA | NA |
| E 14xx Modified Starches | 80.91 | 872.46 | 4016.36 | 1274.82 (1515.28) | 13.10 | 62.97 | 20.66 (29.08) | NA | NA | NA |
| E 300 Ascorbic acid | 73.27 | 4.43 | 32.70 | 8.78 (13.82) | 0.07 | 0.52 | 0.14 (0.22) | NA | NA | NA |
| E 415 Xanthan gum | 69.22 | 198.00 | 1211.60 | 339.91 (461.35) | 3.00 | 19.20 | 5.31 (7.26) | NA | NA | NA |
| E 440 Pectins | 65.87 | 50.23 | 428.26 | 115.71 (188.02) | 0.82 | 6.69 | 1.82 (3) | NA | NA | NA |
| E 471 Mono-and diglycerides of fatty acids | 63.44 | 41.67 | 500.00 | 119.56 (190.65) | 0.64 | 7.62 | 1.83 (2.78) | NA | NA | NA |
| E 407 Carrageenan | 62.51 | 7.26 | 182.86 | 40.01 (69.63) | 0.12 | 2.77 | 0.63 (1.09) | 75 | 0 | 0 |
| E 500 Sodium carbonates | 60.39 | 290.35 | 5100.00 | 1236.59 (2257.99) | 4.67 | 80.65 | 19.24 (32.77) | NA | NA | NA |
| E 412 Guar gum | 57.42 | 70.29 | 921.50 | 216.42 (349.88) | 1.11 | 13.85 | 3.38 (5.42) | NA | NA | NA |
| E 250 Sodium nitrite | 54.22 | 0.03 | 0.71 | 0.17 (0.45) | 0.00 | 0.01 | 0 (0.01) | 0.07 | 0.04 | 0.07 |
| E 450 Diphosphates | 54.03 | 23.99 | 881.53 | 187.08 (354.29) | 0.38 | 13.48 | 2.89 (5.38) | 40 | 0.04 | 0.07 |
| E 202 Potassium sorbate | 50.48 | 0.00 | 66.64 | 13.6 (28.94) | 0.00 | 1.05 | 0.21 (0.44) | 3 | 0.08 | 0.16 |
| E 301 Sodium ascorbate | 37.83 | 0.00 | 25.60 | 5.31 (13) | 0.00 | 0.41 | 0.08 (0.21) | NA | NA | NA |
| E 316 Sodium erythorbate | 35.25 | 0.00 | 23.66 | 4.63 (14.32) | 0.00 | 0.35 | 0.07 (0.21) | 6 | 0 | 0 |
| E 503 Ammonium carbonates | 30.93 | 0.00 | 1530.00 | 261.72 (698.42) | 0.00 | 24.64 | 4.19 (11.15) | NA | NA | NA |
| E 331 Sodium citrates | 30.12 | 0.00 | 285.71 | 62.25 (211.29) | 0.00 | 4.57 | 1.08 (4.02) | NA | NA | NA |
| E 160c Paprika extract, capsanthin, capsorubin | 29.31 | 0.00 | 0.54 | 0.1 (0.31) | 0.00 | 0.01 | 0 (0) | 24 | 0 | 0 |
| E 160a Carotenes | 29.27 | 0.00 | 5.87 | 1 (5.08) | 0.00 | 0.08 | 0.02 (0.08) | NA | NA | NA |
| E 270 Lactic acid | 26.03 | 0.00 | 11.62 | 1.88 (5.9) | 0.00 | 0.19 | 0.03 (0.09) | NA | NA | NA |
| E 410 Locust bean gum | 25.07 | 0.00 | 392.86 | 69.84 (195.5) | 0.00 | 6.30 | 1.11 (3.13) | NA | NA | NA |
| E 422 Glycerol | 24.06 | 0.00 | 1029.28 | 193.53 (918) | 0.00 | 16.36 | 2.89 (11.01) | NA | NA | NA |
| E 252 Potassium nitrate | 23.41 | 0.00 | 0.90 | 0.15 (0.44) | 0.00 | 0.01 | 0 (0.01) | 3.7 | 0 | 0 |
| E 120 Cochineal, Carminic acid, Carmines | 23.29 | 0.00 | 1.11 | 0.32 (1.61) | 0.00 | 0.02 | 0 (0.03) | 5 | 0 | 0 |
| E 224 Potassium metabisulphite | 22.14 | 0.00 | 2.96 | 0.65 (3.24) | 0.00 | 0.05 | 0.01 (0.05) | 0.7 | 0.08 | 0.36 |
| E 160 Carotene | 21.21 | 0.00 | 0.39 | 0.23 (1.84) | 0.00 | 0.01 | 0 (0.03) | NA | NA | NA |
| E 282 Calcium propionate | 21.21 | 0.00 | 66.87 | 10.73 (32.81) | 0.00 | 1.05 | 0.17 (0.51) | NA | NA | NA |
| E 150a Plain caramel | 19.86 | 0.00 | 52.59 | 9.44 (42.37) | 0.00 | 0.78 | 0.15 (0.65) | 300 | 0 | 0 |
| E 451 Triphosphates | 19.32 | 0.00 | 357.14 | 62.46 (239.83) | 0.00 | 5.39 | 0.95 (3.54) | 40 | 0.04 | 0.07 |
| E 420 Sorbitols | 18.97 | 0.00 | 219.78 | 36.96 (140.81) | 0.00 | 3.51 | 0.57 (2.28) | NA | NA | NA |
| E 100 Curcumin | 18.70 | 0.00 | 1.67 | 0.35 (1.8) | 0.00 | 0.03 | 0.01 (0.03) | NA | NA | NA |
| E 160b Annatto, Bixin, Norbixin | 15.23 | 0.00 | 0.30 | 0.05 (0.19) | 0.00 | 0.00 | 0 (0) | 0.3 | 0 | 0 |
| E 150d Sulphite ammonia caramel | 14.58 | 0.00 | 248.21 | 43.8 (218.45) | 0.00 | 3.84 | 0.67 (3.25) | 300 | 0 | 0 |
| E 392 Extracts of rosemary | 14.00 | 0.00 | 3.11 | 0.52 (2.46) | 0.00 | 0.05 | 0.01 (0.04) | 0.3 | 0.44 | 3.08 |
| E 452 Polyphosphates | 13.81 | 0.00 | 147.50 | 30.39 (162.92) | 0.00 | 2.10 | 0.47 (2.45) | 40 | 0.04 | 0.07 |
| E 161b Lutein | 13.50 | 0.00 | 0.02 | 0.59 (8.16) | 0.00 | 0.00 | 0.01 (0.11) | 1 | 0.24 | 1.74 |
| E 950 Acesulfame K | 12.50 | 0.00 | 10.91 | 1.87 (9.3) | 0.00 | 0.17 | 0.03 (0.15) | NA | NA | NA |
| E 341 Calcium phosphates | 12.46 | 0.00 | 107.58 | 22.51 (138.4) | 0.00 | 1.74 | 0.38 (2.38) | 40 | 0.08 | 0.63 |
| E 296 Malic acid | 12.07 | 0.00 | 47.95 | 9.04 (43.33) | 0.00 | 0.73 | 0.13 (0.62) | NA | NA | NA |
| E 262 Sodium acetates | 11.18 | 0.00 | 23.16 | 3.24 (13.99) | 0.00 | 0.35 | 0.05 (0.21) | NA | NA | NA |
| E 162 Beetroot Red, betanin | 9.91 | 0.00 | 1.90 | 0.36 (1.86) | 0.00 | 0.03 | 0.01 (0.03) | NA | NA | NA |
| E 414 Gum arabic (acacia gum) | 9.60 | 0.00 | 104.11 | 31.32 (232.7) | 0.00 | 1.73 | 0.49 (3.61) | NA | NA | NA |
| E 338 Phosphoric acid | 9.37 | 0.00 | 32.57 | 5.48 (28.88) | 0.00 | 0.50 | 0.08 (0.43) | NA | NA | NA |
| E 481 Sodium stearoyl-2-lactylate | 9.10 | 0.00 | 32.48 | 5.83 (32.11) | 0.00 | 0.54 | 0.09 (0.5) | 22 | 0 | 0 |
| E 464 Hydroxypropyl methyl cellulose | 8.64 | 0.00 | 0.09 | 0.02 (0.08) | 0.00 | 0.00 | 0 (0) | NA | NA | NA |
| E 621 Monosodium glutamate | 8.56 | 0.00 | 351.61 | 1999.13 (19574.44) | 0.00 | 5.63 | 30 (275.87) | 30 | 4.35 | 50.23 |
| E 1442 Hydroxy propyl distarch phosphate | 8.18 | 0.00 | 178.57 | 30.14 (153.59) | 0.00 | 2.91 | 0.45 (2.33) | NA | NA | NA |
| E 955 Sucralose | 8.06 | 0.00 | 3.12 | 1.28 (11.23) | 0.00 | 0.05 | 0.02 (0.16) | NA | NA | NA |
| E 260 Acetic acid | 7.94 | 0.00 | 89.29 | 15.47 (82.25) | 0.00 | 1.24 | 0.24 (1.34) | NA | NA | NA |
| E 466 Sodium carboxy methyl cellulose, Cellulose gum | 7.79 | 0.00 | 53.57 | 13.91 (86.77) | 0.00 | 0.85 | 0.22 (1.4) | NA | NA | NA |
| Weighted | | | | | | | |  |  |  |

## APPENDIX 5: Main vector foods for the 50 most frequently consumed food additives in term of number of declarations, NutriNet-Santé cohort, France 2009-2020 (N=106,489)

| **Food additive** | **Main vector foods, confectionary or beverages** |
| --- | --- |
| E 14xx Modified Starches | seasoned grated carrots; quiche lorraine; mixed vegetable soup; light vinaigrette; 0% fat fruit yoghurt, sweetened |
| E 330 Citric acid | button mushroom; jam; light jam; mustard; fruit syrup to be diluted |
| E 322 Lecithins | milk chocolate; dark chocolate; dark chocolate 70% cocoa; omega 3 enriched margarine; chocolate and hazelnut spread |
| E 300 Ascorbic acid | rusk; button mushroom; sweet applesauce; compote without added sugar; chocolate bread |
| E 415 Xanthan gum | seasoned red beets; seasoned grated carrots; mayonnaise; vinaigrette; olive oil vinaigrette |
| E 440 Pectins | jam; light jam; soy fruit dessert cream; lightened thick fresh cream; 0% fat fruit yoghurt, sweetened |
| E 471 Mono;and diglycerides of fatty acids | light margarine 38; 41% fat; omega 3 enriched margarine; wholemeal bread (bread for toast); plain bread (bread for toast); Viennese bread or brioche |
| E 407 Carrageenan | chocolate dessert cream; lightened thick fresh cream; liquid fresh cream; vinaigrette; olive oil vinaigrette |
| E 250 Sodium nitrite | white ham (cooked); quiche lorraine |
| E 412 Guar gum | seasoned red beets; vanilla ice cream; ice cream, other flavor; vinaigrette; 0% fat fruit yoghurt, sweetened |
| E 500 Sodium carbonates | chocolate cookie; breakfast cookie; dry cookie; cookie type small butter; madeleine |
| E 202 Potassium sorbate | seasoned grated carrots; margarine 60% MG; light margarine 38; 41% fat; light margarine enriched in sterols 35% MG; vinaigrette |
| E 450 Diphosphates | chocolate cookie; breakfast cookie; dry cookie; cookie; madeleine |
| E 316 Sodium erythorbate | white ham (cooked) superior; smoked bacon, cooked; quiche lorraine |
| E 301 Sodium ascorbate | white ham (cooked); white ham (cooked) superior; plain bacon, cooked; ham and cheese pizza; quiche lorraine |
| E 160c Paprika extract, capsanthin, capsorubin | surimi stick; mayonnaise; Strasbourg sausage; crab surimi; 0% fat fruit yoghurt, sweetened |
| E 331 Sodium citrates | cappuccino powder; croque; monsieur; light margarine 38-41% fat; diet cola soda; fruit yogurt |
| E 224 Potassium metabisulphite | mayonnaise; mustard; seasoned potato salad; olive oil vinaigrette; light vinaigrette |
| E 503 Ammonium carbonates | chocolate cookie; breakfast cookie; dry cookie; cookie type small butter; snack filled with chocolate or fruit |
| E 160a Carotenes | natural aperitif cookie (cracker type); light margarine 38-41% fat; omega 3 enriched margarine; margarine rich in omega 3 and 6 55-60% MG; vinaigrette |
| E 160 Carotene | low; fat butter 25% fat; vanilla ice cream; margarine 60% MG; light margarine enriched in sterols 35% MG; omega 3 enriched margarine |
| E 410 Locust bean gum | vanilla ice cream; chocolate ice cream; ice cream, other flavor; yoghurt cake; sorbet (water ice) |
| E 950 Acesulfame K | sugar-free chewing gum; sweetener; diet cola soda; 0% MG flavoured yoghurt, sweetened; 0% fat fruit yoghurt, sweetened |
| E 270 Lactic acid | low-fat butter 25% fat; carrot; light margarine 38-41% fat; tiramisu; vinaigrette |
| E 120 Cochineal, Carminic acid, Carmines | 20% fat white cheese with fruits (3% on finished product); yoghurt cake; seasoned potato salad; Strasbourg sausage; tarama |
| E 100 Curcumin | sugar-free chewing gum; flan; badge; lemon pie; 0% fat fruit yoghurt, sweetened |
| E 252 Potassium nitrate | dry cured ham (raw), defatted, defatted; cured ham (raw) type Bayonne, Parma; rosette / spindle; dry sausage |
| E 150d Sulphite ammonia caramel | cola soda; diet cola soda; vinaigrette; olive oil vinaigrette; no added salt vinaigrette |
| E 420 Sorbitols | chocolate cereal bar; fruit cake; sugar-free chewing gum; soft cake with chocolate, chocolate chips or milk filling; madeleine |
| E 951 Aspartame | sugar-free chewing gum; aspartame sweetener; diet cola soda; 0% fat fruit yoghurt, sweetened; 0% fat fruit yoghurt, sweetened |
| E 422 Glycerol | fruit cake; sugar-free chewing gum; chocolate cake; madeleine; gluten-free white bread |
| E 161b Lutein | jelly candy (crocodile type); mayonnaise; low-fat mayonnaise; light vinaigrette; 0% fat fruit yoghurt, sweetened |
| E 392 Extracts of rosemary | mayonnaise; ham and cheese pizza; royal pizza (ham, cheese, mushrooms); industrial mashed potatoes; lemon vinaigrette |
| E 282 Calcium propionate | croque-monsieur; gluten-free white bread; cereal sandwich bread (toast bread); wholemeal bread (bread for toast); plain bread (bread for toast) |
| E 150a Plain caramel | beef bourguignon; 20% fat white cheese with fruits (3% on finished product); poultry ham; slice of oven; baked golden chicken breast; vinaigrette |
| E 451 Triphosphates | cancoillotte; chicken couscous; croque-monsieur; quiche lorraine; Cantonese rice |
| E 452 Polyphosphates | cancoillotte; cappuccino powder; croque-monsieur; Processed cheese 25% MF (12% on finished product); zucchini soup |
| E 338 Phosphoric acid | lemonade; cola soda; decaffeinated cola soda; diet cola soda; diet fruit soda |
| E 160b Annatto, Bixin, Norbixin | vanilla dessert cream; flan; rice pudding; seasoned potato salad; tartiflette |
| E 220 Sulphur dioxide | fruit cake; grape cake; cream sauce; low; fat dressing with no added salt; no added salt vinaigrette |
| E 1442 Hydroxy propyl distarch phosphate | cream dessert; vanilla dessert cream; chocolate dessert cream; 0% MG regular yogurt; sweetened plain whole milk yogurt |
| E 472e Mono; and diacetyl tartaric acid esters of mono; and diglycerides of fatty acids | rusk; breakfast cookie; lightened thick fresh cream; chocolate bread; hollandaise sauce |
| E 621 Monosodium glutamate | reconstituted dehydrated vegetable stock; dehydrated poultry broth, reconstituted; sofas (various trimmings); Aperitif cheese cube; reconstituted meal replacement |
| E 162 Beetroot Red, betanin | strawberry ice cream; 20% fat white cheese with fruits (3% on finished product); yoghurt cake; Savoy cake; badge |
| E 341 Calcium phosphates | 20% fat white cheese with fruits (3% on finished product); chocolate marble cake; fruit muffin; zucchini soup; chocolate pie |
| E 476 Polyglycerol polyricinoleate | low-fat butter 15% fat; low-fat sweet butter; Eskimo or vanilla stick; Eskimo or stick, other flavor; light margarine 38-41% fat |
| E 955 Sucralose | sugar-free candy; syrup 0% sugar to be diluted; diet soda; tonic soda; sucralose |
| E 163 Anthocyanins | jelly candy (crocodile type); Christmas log; 20% fat white cheese with fruits (3% on finished product); 40% fat white cheese with fruits (6% on finished product); 0% fat fruit yoghurt, sweetened |
| E 296 Malic acid | Cereal cookie; dry cookie; jelly candy (crocodile type); sugar-free chewing gum; salmon sushi |
| E 466 Sodium carboxy methyl cellulose, Cellulose gum | low-fat butter 15% fat; low-fat sweet butter; sugar-free chewing gum; clafoutis; lightened thick fresh cream |

## APPENDIX 6a: Correlation between NMF components and food additive intakes, NutriNet-Santé cohort, France 2009-2020 (N=106,489)

| **Food additive** | **Component 1** | **Component 2** | **Component 3** | **Component 4** | **Component 5** |
| --- | --- | --- | --- | --- | --- |
| E 14xx Modified Starches | 0,19 | 0,99 | 0,00 | 0,16 | 0,01 |
| E 330 Citric acid | 0,35 | 0,30 | 0,23 | 0,06 | 0,06 |
| E 322 Lecithins | 0,11 | 0,05 | 0,41 | 0,21 | 0,09 |
| E 440 Pectins | 0,17 | 0,31 | 0,02 | 0,09 | 0,22 |
| E 300 Ascorbic acid | 0,18 | 0,09 | 0,11 | 0,05 | 0,03 |
| E 415 Xanthan gum | 0,86 | 0,24 | 0,07 | 0,17 | 0,04 |
| E 471 Mono-and diglycerides of fatty acids | 0,35 | 0,05 | 0,26 | 0,27 | 0,09 |
| E 407 Carrageenan | 0,12 | 0,11 | 0,05 | 0,98 | 0,04 |
| E 202 Potassium sorbate | 0,56 | 0,11 | 0,06 | 0,19 | 0,07 |
| E 412 Guar gum | 0,84 | 0,10 | 0,01 | 0,09 | 0,06 |
| E 250 Sodium nitrite | 0,29 | 0,09 | 0,10 | 0,08 | 0,07 |
| E 500 Sodium carbonates | 0,04 | 0,06 | 0,86 | 0,07 | 0,02 |
| E 450 Diphosphates | 0,12 | 0,05 | 0,61 | 0,10 | 0,02 |
| E 950 Acesulfame K | 0,06 | 0,03 | 0,02 | 0,05 | 0,82 |
| E 160a Carotenes | 0,13 | 0,04 | 0,04 | 0,08 | 0,10 |
| E 331 Sodium citrates | 0,10 | 0,08 | 0,06 | 0,10 | 0,82 |
| E 951 Aspartame | 0,02 | 0,03 | -0,01 | 0,02 | 0,69 |
| E 301 Sodium ascorbate | 0,16 | 0,06 | 0,11 | 0,06 | 0,08 |
| E 160c Paprika extract, capsanthin, capsorubin | 0,25 | 0,15 | 0,04 | 0,12 | 0,17 |
| E 316 Sodium erythorbate | 0,24 | 0,09 | 0,06 | 0,04 | 0,03 |
| E 224 Potassium metabisulphite | 0,34 | 0,04 | 0,07 | 0,02 | 0,03 |
| E 503 Ammonium carbonates | 0,02 | -0,02 | 0,79 | 0,04 | 0,03 |
| E 270 Lactic acid | 0,17 | 0,07 | 0,04 | 0,13 | 0,01 |
| E 150d Sulphite ammonia caramel | 0,05 | -0,04 | 0,05 | 0,03 | 0,92 |
| E 100 Curcumin | 0,07 | 0,20 | -0,03 | 0,21 | 0,20 |
| E 120 Cochineal, Carminic acid, Carmines | 0,15 | 0,06 | 0,05 | 0,04 | 0,06 |
| E 252 Potassium nitrate | 0,08 | -0,02 | 0,10 | 0,01 | 0,04 |
| E 422 Glycerol | 0,08 | 0,07 | 0,33 | 0,04 | 0,02 |
| E 282 Calcium propionate | 0,16 | 0,01 | 0,09 | 0,15 | 0,04 |
| E 420 Sorbitols | 0,07 | 0,01 | 0,22 | 0,05 | 0,03 |
| E 150a Plain caramel | 0,16 | 0,04 | 0,05 | 0,10 | 0,03 |
| E 161b Lutein | 0,06 | 0,06 | 0,03 | 0,01 | 0,08 |
| E 338 Phosphoric acid | 0,04 | -0,04 | 0,05 | 0,03 | 0,92 |
| E 451 Triphosphates | 0,35 | 0,09 | 0,06 | 0,10 | 0,02 |
| E 452 Polyphosphates | 0,25 | 0,08 | 0,04 | 0,23 | 0,03 |
| E 476 Polyglycerol polyricinoleate | 0,06 | 0,00 | 0,11 | 0,07 | 0,04 |
| E 392 Extracts of rosemary | 0,09 | 0,01 | 0,07 | 0,05 | 0,05 |
| E 955 Sucralose | 0,04 | 0,01 | 0,01 | 0,02 | 0,14 |
| E 160b Annatto, Bixin, Norbixin | 0,11 | 0,10 | 0,04 | 0,22 | 0,02 |
| E 1442 Hydroxy propyl distarch phosphate | 0,02 | 0,00 | 0,01 | 0,40 | 0,05 |
| E 220 Sulphur dioxide | 0,01 | 0,01 | 0,05 | 0,00 | 0,01 |
| E 472e Mono- and diacetyl tartaric acid esters of mono- and diglycerides of fatty acids | 0,02 | 0,00 | 0,25 | 0,05 | 0,03 |
| E 621 Monosodium glutamate | 0,02 | 0,05 | 0,01 | 0,00 | -0,01 |
| E 306 Tocopherol-rich extract | 0,06 | 0,00 | 0,03 | 0,02 | 0,03 |
| E 341 Calcium phosphates | 0,01 | 0,04 | 0,06 | 0,03 | 0,02 |
| E 481 Sodium stearoyl-2-lactylate | 0,05 | -0,01 | 0,11 | 0,09 | 0,04 |
| E 150c Ammonia caramel | 0,02 | 0,05 | 0,05 | 0,04 | 0,01 |
| E 163 Anthocyanins | 0,08 | 0,06 | 0,04 | 0,10 | 0,09 |
| E 385 Calcium disodium ethylene diamine tetra-acetate (Calcium disodium EDTA) | 0,05 | -0,01 | 0,02 | 0,01 | 0,01 |
| E 960 Steviol glycosides | 0,01 | 0,02 | 0,00 | 0,03 | 0,02 |
| E 340 Potassium phosphates | 0,00 | 0,00 | 0,02 | 0,02 | 0,03 |
| E 442 Ammonium phosphatides | 0,08 | -0,04 | 0,02 | 0,15 | 0,02 |
| E 334 Tartaric acid (L(+)-) | 0,02 | 0,01 | 0,04 | 0,00 | 0,00 |
| E 200 Sorbic acid | 0,03 | 0,01 | 0,07 | 0,01 | 0,00 |
| E 223 Sodium metabisulphite | 0,12 | 0,07 | 0,00 | -0,01 | -0,01 |
| E 211 Sodium benzoate | 0,03 | 0,00 | 0,00 | 0,01 | 0,06 |
| E 133 Brilliant Blue FCF | 0,06 | 0,00 | 0,03 | 0,01 | 0,03 |
| E 339 Sodium phosphates | 0,01 | 0,04 | 0,03 | 0,02 | 0,02 |
| E 172 Iron oxides and hydroxides | 0,19 | 0,05 | -0,01 | 0,00 | -0,01 |
| E 475 Polyglycerol esters of fatty acids | 0,03 | -0,02 | 0,10 | 0,06 | 0,04 |
| E 954 Saccharins | 0,01 | 0,02 | 0,00 | 0,00 | 0,03 |
| E 131 Patent Blue V | 0,02 | 0,00 | 0,04 | 0,02 | 0,06 |
| E 150 Caramel | 0,01 | 0,00 | 0,00 | 0,00 | 0,01 |
| E 473 Sucrose esters of fatty acids | 0,02 | 0,00 | 0,06 | 0,01 | 0,02 |
| E 102 Tartrazine | 0,06 | 0,01 | 0,02 | 0,01 | 0,02 |
| E 445 Glycerol esters of wood rosins | 0,03 | 0,02 | 0,01 | 0,02 | 0,10 |
| E 234 Nisin | 0,04 | 0,00 | 0,01 | 0,05 | 0,01 |
| E 304 Fatty acid esters of ascorbic acid | -0,01 | 0,11 | 0,02 | 0,01 | 0,02 |
| E 160e Beta-apo-8'-carotenal (C 30) | 0,02 | 0,00 | 0,03 | 0,01 | 0,03 |
| E 952 Cyclamates | 0,01 | 0,02 | 0,00 | 0,01 | 0,04 |
| E 472 Esters of mono- and diglycerides | 0,02 | 0,02 | 0,03 | 0,01 | 0,02 |
| E 320 Butylated hydroxyanisole (BHA) | 0,01 | 0,01 | 0,01 | 0,00 | 0,01 |
| E 222 Sodium hydrogen sulphite | 0,04 | 0,01 | 0,03 | 0,01 | 0,01 |
| E 110 Sunset Yellow FCF/Orange Yellow S | 0,02 | 0,01 | 0,00 | 0,01 | 0,00 |
| E 251 Sodium nitrate | 0,02 | 0,00 | 0,02 | 0,01 | 0,01 |
| E 212 Potassium benzoate | 0,01 | 0,00 | 0,01 | 0,00 | 0,06 |
| E 249 Potassium nitrite | 0,03 | 0,01 | 0,04 | 0,01 | 0,01 |
| E 321 Butylated hydroxytoluene (BHT) | 0,00 | 0,00 | 0,01 | 0,00 | 0,01 |
| E 104 Quinoline Yellow | 0,00 | 0,00 | 0,00 | 0,00 | 0,06 |
| E 280 Propionic acid | 0,04 | 0,00 | 0,01 | 0,00 | 0,01 |
| E 482 Calcium stearoyl-2-lactylate | 0,01 | 0,00 | 0,02 | 0,00 | 0,00 |
| E 302 Calcium ascorbate | 0,01 | 0,00 | 0,01 | 0,00 | 0,00 |
| E 444 Sucrose acetate isobutyrate | 0,01 | 0,01 | 0,00 | 0,01 | 0,02 |
| E 242 Dimethyl dicarbonate | 0,00 | 0,01 | 0,00 | 0,00 | 0,00 |
| E 129 Allura Red AC | 0,00 | 0,00 | 0,01 | 0,00 | 0,01 |
| E 962 Salt of aspartame-acesulfame | 0,00 | 0,00 | 0,01 | 0,00 | 0,01 |
| E 132 Indigotine, Indigo carmine | 0,00 | 0,00 | 0,00 | 0,00 | 0,00 |
| E 319 Tertiary-butyl hydroquinone (TBHQ) | 0,00 | 0,00 | 0,03 | 0,00 | 0,00 |
| E 285 Sodium tetraborate (borax) | -0,01 | 0,00 | 0,00 | 0,00 | 0,00 |
| E 435 Polyoxyethylene sorbitan monostearate (polysorbate 60) | 0,00 | 0,00 | 0,00 | 0,01 | 0,00 |

## APPENDIX 6b: Means of scaled NMF components, by clusters of participants, NutriNet-Santé cohort, France 2009-2020 (N=106,489)

| Cluster | Component 1 | Component 2 | Component 3 | Component 4 | Component 5 |
| --- | --- | --- | --- | --- | --- |
| 1 | -0,10 | -0,15 | **2,21** | -0,08 | -0,06 |
| 2 | -0,27 | **1,62** | -0,31 | -0,20 | -0,16 |
| 3 | 0,08 | 0,11 | -0,09 | **2,39** | -0,05 |
| 4 | **1,83** | 0,13 | -0,17 | -0,05 | -0,04 |
| 5 | 0,03 | -0,06 | -0,02 | 0,02 | **4,69** |
| 6 | -0,35 | -0,48 | -0,27 | -0,30 | -0,16 |

## REFERENCES

1. European Commission. Database. *Food Safety - European Commission* https://ec.europa.eu/food/safety/food_improvement_agents/additives/database_en (2016).

2. *Codex General Standard for Food Additives (GSFA, Codex STAN 192-1995)*. http://www.fao.org/fao-who-codexalimentarius/sh-proxy/en/?lnk=1&url=https%253A%252F%252Fworkspace.fao.org%252Fsites%252Fcodex%252FStandards%252FCODEX%2BSTAN%2B192-1995%252FCXS_192e.pdf (2018).

3. Paatero, P. & Tapper, U. Positive matrix factorization: A non-negative factor model with optimal utilization of error estimates of data values. *Environmetrics* **5**, 111–126 (1994).

4. Lee, D. D. & Seung, H. S. Learning the parts of objects by non-negative matrix factorization. *Nature* **401**, 788–791 (1999).

5. Zetlaoui, M., Feinberg, M., Verger, P. & Clémençon, S. Extraction of Food Consumption Systems by Nonnegative Matrix Factorization (NMF) for the Assessment of Food Choices. *Biometrics* **67**, 1647–1658 (2011).

6. Hoyer, P. O. Non-negative matrix factorization with sparseness constraints. *arXiv:cs/0408058* (2004).

7. Brunet, J.-P., Tamayo, P., Golub, T. R. & Mesirov, J. P. Metagenes and molecular pattern discovery using matrix factorization. *PNAS* **101**, 4164–4169 (2004).
